# Heat‐Evolved Microalgae (Symbiodiniaceae) Are Stable Symbionts and Influence Thermal Tolerance of the Sea Anemone *Exaiptasia diaphana*

**DOI:** 10.1111/1462-2920.70011

**Published:** 2025-01-21

**Authors:** Wing Yan Chan, Rumi Sakamoto, Talisa Doering, Vinod K. Narayana, David P. De Souza, Malcolm J. McConville, Madeleine J. H. van Oppen

**Affiliations:** ^1^ Department of Biochemistry and Pharmacology Bio21 Institute of Molecular Science and Biotechnology, the University of Melbourne Parkville Victoria Australia; ^2^ Australian Institute of Marine Science Townsville Queensland Australia; ^3^ School of Biosciences The University of Melbourne Parkville Victoria Australia; ^4^ Metabolomics Australia, Bio21 Institute of Molecular Science and Technology The University of Melbourne Parkville Victoria Australia

**Keywords:** coral bleaching, *Exaiptasia diaphana*, experimental evolution, metabolomics, Symbiodiniaceae, symbiosis

## Abstract

Symbiotic cnidarians, such as sea anemones and corals, rely on their mutualistic microalgal partners (Symbiodiniaceae) for survival. Marine heatwaves can disrupt this partnership, and it has been proposed that introducing experimentally evolved, heat‐tolerant algal symbionts could enhance host thermotolerance. To test this hypothesis, the sea anemone *Exaiptasia diaphana* (a coral model) was inoculated with either the heterologous wild type or heat‐evolved algal symbiont, *Cladocopium proliferum*, and homologous wild‐type *Breviolum minutum*. The novel symbioses persisted for 1.5 years and determined holobiont thermotolerance during a simulated summer heatwave. Anemones hosting SS8, one of the six heat‐evolved strains tested, exhibited the highest thermotolerance. Notably, anemones hosting the wild‐type *C. proliferum* (WT10) were the second most thermally tolerant group, whereas anemones hosting the heat‐evolved SS5 or SS9 strains were among the most thermosensitive. Elevated temperatures led to an increase in the levels of many amino acids and a decrease in tricarboxylic acid (TCA) metabolites in all anemone hosts, potentially indicating an increase in autophagy and a reduction in energy and storage production. Some consistent differences were observed in changes in metabolite levels between anemone groups in response to elevated temperature, suggesting that the algal symbiont influenced host metabolome and nutritional budget.

## Graphical abstract

Heat‐evolved photosymbionts have maintained long‐term symbiosis with sea anemones, impacting their thermotolerance and metabolome in distinct ways under simulated summer heatwave conditions. Notably, one of the ten heat‐evolved strains (SS8) promoted high anemone thermotolerance. Our finding suggests that heat stress may increase autophagy and decrease energy and storage production within the anemone host.

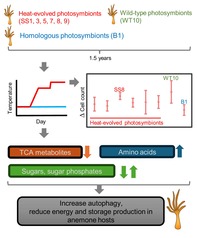

## 1 Introduction

Tropical coral reefs support up to ~30% of all named marine life (Fisher et al. 2015) and are of enormous economic importance, supporting major industries such as tourism and fisheries, while also protecting our coastlines from erosion (Deloitte Access Economics 2017). Scleractinian corals are nutritionally dependent on their photosymbionts—microalgae in the dinoflagellate family of Symbiodiniaceae (Davy, Allemand, and Weis 2012; Quigley et al. 2018). Under non‐stress conditions, corals provide essential inorganic substrates to Symbiodiniaceae and in return, receive > 90% of their net fixed carbon from the symbiont (Muscatine et al. 1984). Increased sea surface temperatures can disrupt this symbiotic relationship, resulting in coral bleaching and starvation of the coral host (Hoegh‐Guldberg 1999). Marine heatwaves are one of the major driving forces of coral population decline globally.

There are several hypotheses on the cellular mechanism underpinning coral bleaching. One hypothesis postulates that elevated temperature and irradiance damage the symbionts' photosystem, leading to an overproduction of toxic reactive oxygen species (ROS) (Weis 2008; Szabó, Larkum, and Vass 2020). The excess ROS can diffuse into the host cells and damage macromolecules, which triggers a cellular cascade that leads to bleaching. Another hypothesis suggests that the mechanisms of coral bleaching are linked to Symbiodiniaceae switching to parasitism (Baker et al. 2018). Under heat stress, amino acid catabolism from the host results in NH_4_^+^ release; this relieves the algal symbiont from nitrogen limitation (commonly experienced in hospite), encourages their proliferation, but reduces carbon translocation to the host and exacerbates the state of host starvation (Rädecker et al. 2021). It is possible that, in a healthy symbiosis, the release of photosynthates from the symbiont could mimic the digestion of prey and thereby arrest the maturation of the phagosome. If photosynthate translocation were to be reduced, this might facilitate phagosome maturation, potentially leading to the digestion and expulsion of the symbiont (Hill and Hill 2012). Additionally, symbiont proliferation increases cellular phosphorus demand. Phosphate resources are subsequently prioritised to rescue the most vital cellular functions, which alters the composition of symbiont thylakoid membranes, reduces their threshold for heat‐ and light‐induced bleaching (Wiedenmann et al. 2013) and increases the production of damaging ROS (Smith, Suggett, and Baker 2005).

As the physiological properties of Symbiodiniaceae play a major role in host bleaching and thermal tolerance (Berkelmans and van Oppen 2006; Quigley et al. 2018; Palacio‐Castro et al. 2023) it has been proposed that the thermotolerance of the coral host could be enhanced by manipulating their Symbiodiniaceae community via experimental evolution (van Oppen et al. 2015, 2017; Nitschke et al. 2024). In this approach, a monoclonal culture of the Symbiodiniaceae species *Cladocopium proliferum* (Beltrán et al. 2021) was isolated from Great Barrier Reef (GBR) *Acropora tenuis* and experimentally evolved to adapt to a high temperature of 31°C (Chakravarti, Beltran, and van Oppen 2017). In vitro, these experimentally‐evolved strains (hereafter referred to as “heat‐evolved strains”) were able to maintain positive growth and generated less ROS than wild‐type strains under elevated temperature (Buerger et al. 2020). In hospite, three out of the 10 heat‐evolved strains (SS1, 7 and 8) enhanced thermotolerance of coral larvae, juveniles and adults compared to wild‐type strain(s) (Buerger et al. 2020; Quigley and van Oppen 2022; Chan et al. 2023; Quigley et al. 2023), but other heat‐evolved strains did not (Buerger et al. 2020). These promising results suggest that heat‐evolved Symbiodiniaceae could potentially be valuable resources for reef restoration. Prior to implementation, however, more coral/cnidarian species should be tested and the mechanisms of how heat‐evolved Symbiodiniaceae influence host thermotolerance need to be examined.

In the study, we investigated whether heat‐evolved Symbiodiniaceae enhanced the thermotolerance of *Exaiptasia diaphana* (sea anemones), an important experimental model to study cnidarian‐dinoflagellate interactions (e.g., Hillyer, D.A. Dias, et al. 2017; Matthews et al. 2018; Gabay et al. 2019). We also undertook an initial study to identify the major changes in metabolism of different Symbiodiniaceae, *E. diaphana* combinations in response to heat stress responses. Subsets of anemones were chemically bleached to remove their native symbionts and reinoculated with six heterologous (i.e., non‐native) *C. proliferum* heat‐evolved strains (SS1, 7, or 8: which conferred enhanced bleaching tolerance to corals; SS3, 5 or 9: which did not confer enhanced tolerance to corals) (Buerger et al. 2020); one wild‐type *C. proliferum* strain (WT10); and one homologous *B. minutum* strain (B1) (Table S1). Symbiosis was maintained for 1.5 years at ambient temperature, before the holobiont was exposed to a simulated heatwave. Physiological parameters (photochemical efficiency, Symbiodiniaceae cell density, respiration and gross photosynthesis) were measured, and the host metabolome was analysed using targeted liquid‐chromatography–mass spectrometry (LC–MS).

## 2 Materials and Methods

### 2.1 Experimental Organisms and Symbiodiniaceae Identity

Experimental anemones (*E. diaphana* genotype AIMS4) were sourced from the culture collection at the Microbial Symbiosis Lab at the University of Melbourne. These sea anemones were originally collected from the central GBR and their homologous algal symbiont was *B. minutum* (Dungan et al. 2020). Aposymbiotic (i.e., free of algal symbionts) *E. diaphana* was obtained via a modified menthol/diuron bleaching method (Matthews et al. 2016). The eight anemone groups were previously produced via inoculation with three heterologous heat‐evolved strains ( *C. proliferum* ) that are known to confer enhanced bleaching tolerance to corals (SS1, SS7 or SS8), and three heat‐evolved strains that did not confer enhanced tolerance to corals (SS3, 5 or 9). In addition, wild‐type *C. proliferum* and the homologous *B. minutum* were used for inoculation of anemones (Tsang Min Ching et al. 2022) (Table S1).

During the 1.5 years of growing period following inoculation, each anemone group was kept in separate 1 L containers (< 40 individuals per container, four containers per anemone groups) filled with reconstituted seawater (RSW; Red Sea Salt and reverse osmosis water, 34 ppt salinity). The tanks were kept in incubators at 27°C under a light intensity of 30 μmol photons m^−2^ s^−1^ (12 h light:12 h dark). Compared to corals, *E. diaphana* from the GBR is highly sensitive to light even under ambient temperatures. The chosen light intensity represents the highest intensity that this anemone genotype can acclimatise to (Dungan, Hartman, et al. 2022; Doering et al. 2023a). Anemones were fed with freshly hatched *Artemia salina* up to four times a week, and the tanks were cleaned and refilled with freshly made RSW at the end of each feeding day. Prior to the experiment, ITS2 metabarcoding was conducted to confirm that the anemones still hosted the Symbiodiniaceae strains they were inoculated with. Two samples per anemone group were randomly selected, homogenised, snap frozen and stored at −80°C until further processing (Appendix Methods S1). DNA extraction, PCR amplification and library preparation were conducted following Tsang Min Ching et al. (2022) (Appendix Methods S1). The library was sequenced with Illumina MiSeq v3 at the Walter and Eliza Hall Institute, and raw sequences were analysed in SymPortal (Hume et al. 2019).

### 2.2 Experimental Design

**FIGURE 1:**
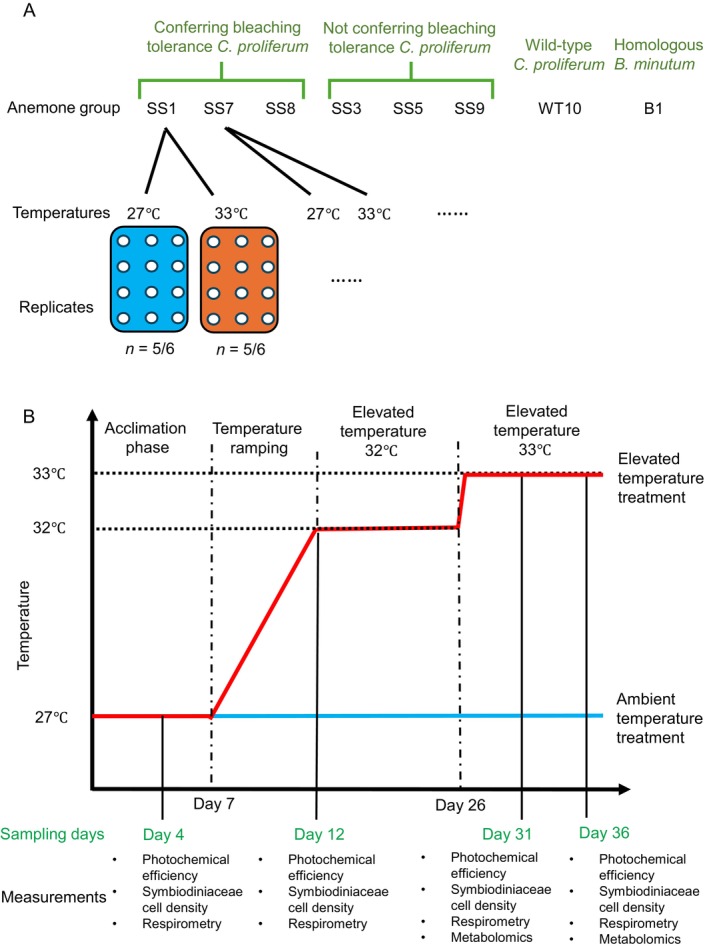
Experimental design and timeline. (A) The eight anemone groups in this study were *Exaiptasia diaphana* inoculated with: Heterologous heat‐evolved *Cladocopium proliferum* that is known to confer enhanced bleaching tolerance to corals (SS1, SS7 or SS8); heat‐evolved *C. proliferum* that did not confer tolerance (SS3, SS5 or SS9); wild‐type *C. proliferum* ; the homologous *Breviolum minutum*. (B) Experimental temperatures, sampling timeline and measurements throughout the experiment.

A total of 988 *E. diaphana* were used for the experiment (SS1: *n* = 132, SS3: *n* = 120, SS5: *n* = 120, SS7: *n* = 120, SS8: *n* = 128, SS9: *n* = 120, WT10: *n* = 130, B1: *n* = 118). There was no visual difference in size between anemone groups and the averaged oral disk diameter of the anemones was approximately 6 mm. Each anemone was transferred into an individual well of a 12‐well‐plate (*n* = 86 plates total, *n* = 10–12 plates per Symbiodiniaceae strain) and allowed to acclimatise at 27°C for 6 days (Figure 1). The plates were evenly and randomly distributed into two temperature treatments (ambient: 27°C; elevated up to 33°C; four incubators total) (Figure 1). Each plate was individually kept in a sealed plastic container, to minimise the risk of cross‐contamination between Symbiodiniaceae strains. To ensure that the anemones received even light intensity and temperature, the location of the containers was randomised every day between incubators and shelves. Light was provided at an intensity of 30 μmol photons m^−2^ s^−1^ (12 h:12 h, light: dark). After the end of acclimation (Day 7), the temperature of the elevated treatment was increased 1°C per day until reaching 32°C (Day 12) (Figure 1B). Since no bleaching was observed on Day 26, the temperature was further increased from 32°C to 33°C on Day 26 and kept until the end of the experiment (Day 36). Anemones were fed *A. salina* every 4 days, and the wells were cleaned and refilled with fresh RSW on the same day of cleaning.

Photochemical efficiency of all anemone groups was measured approximately every 2 days, to guide sampling according to anemone bleaching responses. Symbiodiniaceae cell density was measured for all anemone groups on Day 4 (during acclimation), Day 12 (when elevated temperature reached 32°C), Day 31 (when bleaching was observed, elevated temperature was 33°C) and Day 36 (end of experiment), while gross photosynthesis and respiration rates were measured for four selected anemone groups: SS1‐ and SS8‐anemones (heterologous, heat‐evolved), WT10‐anemones (heterologous, wild‐type) and B1‐anemones (homologous) (Figure 1B). Samples for host metabolome analysis were guided by the physiological data and collected on Day 31 and 36, including the most thermally tolerant (SS8‐anemones) and thermally sensitive (SS9‐anemones) anemone groups hosting heat‐evolved strains, as well as WT10‐anemones hosting the wild‐type strain.

### 2.3 Photochemical Efficiency and Symbiodiniaceae Cell Counts

The maximum quantum yield of photosystem II (Fv/Fm) of the symbionts within hosts was measured via imaging pulse amplitude modulation (PAM) fluorometry (IMAG‐MAX/L, Waltz, Germany) as a proxy for anemone heat stress. Unlike diving PAM where measurements are taken underwater one at a time, the imaging PAM operates on land and multi areas‐of‐interest can be measured per imaging run. In this study, sixteen 12‐well plates (i.e., 12 samples × 8 anemone groups × 2 temperatures, *n* = 192 total) were measured consistently at 2–3 h after the commencement of the morning light cycle. Each imaging run measured one plate and with three technical replicates per anemone. The anemones remained submerged in seawater (as per their normal growing conditions) during measurements. The plates were dark‐adapted for 30 min and measured with the following parameters: measuring light intensity: 3, frequency: 1, actinic light intensity: 3, gain: 2, damping: 2 and saturation pulse intensity: 8. Photochemical efficiency was measured every 2 days, except on Day 32, where measurements were taken on Day 31 instead. For Symbiodiniaceae cell counts, thawed anemone samples (*n* = 4 per treatment) were homogenised, vortexed and pipetted up and down for mixing (Appendix Method S2). Ten μL of the samples were measured in a Countess II FL automated cell counter, gated by size, shape and fluorescence. Four technical replicates were measured per sample. The replicates were averaged and normalised to host protein content (Appendix Methods S2) (Bradford 1976).

### 2.4 Gross Photosynthesis and Respiration

Gross photosynthesis and respiration rates were measured by oxygen production/consumption rates over time, using the FireSting O_2_ optical oxygen sensors (PyroScience, Germany). The sensors were set up in a specific incubator with the same light intensity and the temperature as in the respective experimental incubators. The day before the measurement, anemones were transferred into 12‐well plates with a mesh disk and a strip, which can be moved to the respirometry vials without touching the anemones. Respirometry vials were assembled under RSW to avoid bubble formation. On each sampling day, anemones were transferred to the respirometry vials, containing 14.88 mL of RSW that was preheated to the respective treatment temperature, a stage and a magnetic stirrer underneath it, and a lid with the oxygen sensor attached (Figure S1). After connecting the oxygen sensors to the oxygen electrodes and switching on the magnetic stirrers for each vial, anemones were acclimated inside the incubator in the dark for 30 min. Subsequently, oxygen utilisation as a proxy for respiration rate was measured for an hour in the dark, then the light was switched on to measure oxygen production as a proxy for the net photosynthesis rate for another hour. A total of 32 anemones were measured on a sampling day (4 samples × 4 anemone groups × 2 temperatures). Two blanks (i.e., vials filled with RSW without an anemone) were included per respirometry run to calculate background signals.

The *respR* package was used to determine the change in O_2_ concentrations over time. First, raw data were blank corrected and normalised by the water volume in the vials (14.88 mL). Gross photosynthesis rate was calculated as: *gross photosynthesis = net photosynthesis + |respiration|*, where | | indicates the absolute value. To normalise the gross photosynthesis and respiration data, anemones used for respirometry were collected to analyse host protein content and Symbiodiniaceae cell density (Appendix method S2). Gross photosynthesis rates were then normalised by Symbiodiniaceae cell counts, whereas respiration rates were normalised to host protein content.

### 2.5 Host Metabolite Extraction

A total of 96 samples (8 samples × 3 anemone groups × 2 temperatures × 2 timepoints) were randomly selected for host metabolome analysis. Samples were very briefly rinsed with Milli‐Q water to remove salt, and snap‐frozen in liquid nitrogen and stored at −80°C. Each sample was transferred to a Cryomill tube (MP Biomedical) with 500 μL of 4°C Milli‐Q water, and homogenised (3 × 30 s pulses at 3620 rcf, 45 s interval between pulses) in a pre‐chilled (< 4°C) Cryomill (Precellys24, Bertin Technologies, France). The host and symbiont fractions were separated by centrifugation (16,000 rcf, 10 min, 4°C). The host component (supernatant) was transferred to a new set of pre‐weighted Eppendorf tubes, which were freeze‐dried overnight, weighed, and stored at −80°C until further processing. Freeze‐dried host samples were suspended in 50% MeOH (500 μL, < 4°C) containing internal standards (^13^C_6_ Sorbitol, ^13^C_5_, ^15^N Valine, ^13^C_6_ leucine, 4 μM). Samples were sonicated in a cold‐water bath for 10 min and centrifuged (14,000 rcf, 20 min, 4°C) to pellet insoluble debris, and the supernatant was transferred to fresh Eppendorf tubes. The remaining pellet was re‐extracted with a further 500 μL of cold 50% MeOH, sonicated (10 min in cold water bath) and centrifuged (14,000 rcf, 20 min, 4°C). The supernatant was added to the previous tubes, and polar metabolites were analysed using LC–MS as per (Kong et al. 2021).

Briefly, chromatographic separation of samples (7 μL) was achieved on a SeQuant ZIC‐pHILIC column (150 × 460 mm) using an Agilent 1200 series liquid chromatography system (Agilent Technologies, Santa Clara, USA). Polar metabolites were detected using a Agilent Technologies 6545B series Quadrupole Time of Flight (TOF) mass spectrometer with published acquisition parameters (Stewart et al. 2017; Kong et al. 2021). Targeted data matrices (peak area integration) were generated using MassHunter Quantitative Analysis software (version B.10.00, Agilent technologies), ProFinder (version B.08.00, Agilent technologies) and the Metabolomics Australia in‐house data processing pipeline (Stewart et al. 2017). Metabolite identification was based on the retention time and molecular mass matching to respective authentic standards. Note that disaccharides (e.g., cellobiose, sucrose, trehalose) have very similar LC retention times and have been denoted as disaccharide a–d. Similarly, multiple sugar alcohols (e.g., galactitol, mannitol, sorbitol) and C18: 1 fatty acids (e.g., elaidic acid, oleic acid, vaccenic acid) were identified but not unequivocally annotated. Pooled quality control samples (an aliquot of each sample pooled together) were used and incorporated every five samples in the run.

### 2.6 Statistical Analysis—Physiological Measurements

Statistical analyses of the photochemical efficiency, Symbiodiniaceae cell counts and respirometry data were performed in RStudio (Version 4.1.2) (R Core Team 2021). Each dataset was first tested for normality and homogeneity of variance, and proceeded with a non‐parametric test if necessary. For photochemical efficiency (Fv/Fm, *n* = 12 per anemone group per temperature), measurements were omitted when their *F*_0_ (i.e., the minimum background fluorescence) was < 0.1; as it indicates photoinhibition in the organism and will artificially inflate the Fv/Fm value (Bhagooli et al. 2021). These omitted measurements occurred in a few anemone groups towards the end of the experiment, with the lowest *n* = 4 (Table S2). The non‐parametric Mann–Whitney Wilcoxon test was used to test the effect of temperature on photochemical efficiency within a specific anemone group at one time point. This analysis focused on identifying the time point when a significant difference in photochemical efficiency was first detected in an anemone group between temperature. The thermotolerance of anemone groups were then ranked according to this (i.e., a group was considered less thermally tolerant, when a significant drop in photochemical efficiency occurred earlier in the experiment).

**FIGURE 2:**
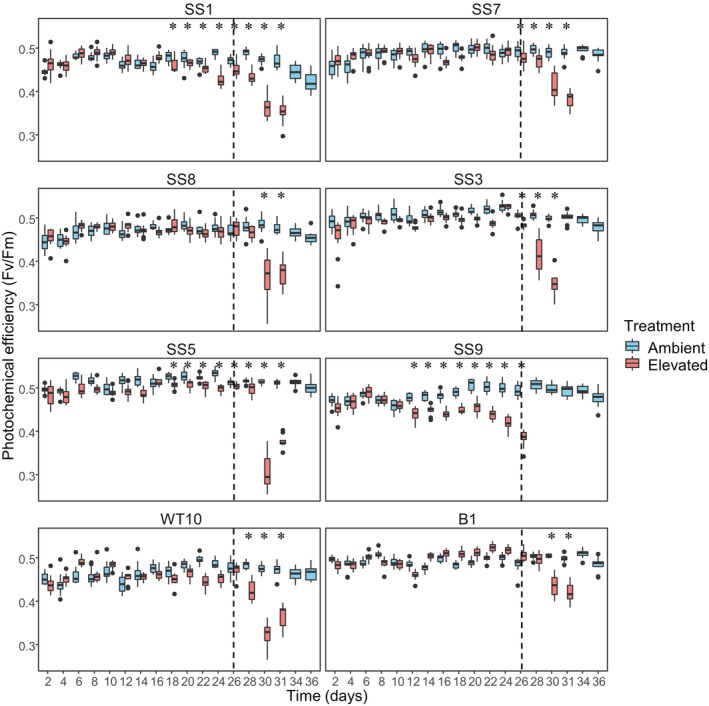
Photochemical efficiency and thermotolerance rank of anemone groups for this trait. Photochemical efficiency (maximum quantum yield of the photosystem II, Fv/Fm) during the experiment. * indicates significant differences between temperatures. *n* = 12 per anemone group in both temperatures up to Day 26. By Day 28, some anemones started to experience photoinhibition, where their *F*_0_ values dropped below 0.1. These measurements were excluded as they will artificially Fv/Fm (Bhagooli et al. 2021). For this reason, a few of the later time points under elevated temperatures did not have Fv/Fm data. Most plotted elevated temperature values from Day 28 had *n* = 11–12, with the exceptions shown in Table S2. The dash line indicates when the elevated temperature treatment reached 33°C.

For Symbiodiniaceae cell counts, the data were first normalised by host protein content and two statistical analyses were used. The first analysis tested the effect of temperature on cell density change (%) within a specific anemone group at one time point, using the non‐parametric Mann–Whitney Wilcoxon test. The cell density change was calculated as (Cell_day*x*_/Cell_day4_) × 100, where *x* referred to a specific time point (Day 12, 31 or 36) and Day 4 was the cell density during acclimatisation (i.e., before temperature ramping). The second analysis tested the effect of anemone groups on cell density change, which was calculated as Cell_Day36_−Cell_Day4_. Generalised linear models with one fixed factorial (anemone group) and a quasi‐binomial distribution was used for this analysis. Model assumptions were visually inspected and confirmed. For the respirometry analysis, Kruskal–Wallis rank tests were conducted to test the effect of anemone groups within the same temperature at one time point; and Mann–Whitney Wilcoxon tests were used to test the effect of temperature within a specific anemone group at one time point. The *p* values of multiple pairwise comparison were adjusted with the Benjamini–Hochberg method (Benjamini and Hochberg 1995).

### 2.7 Statistical Analysis—Metabolomics

Raw host data were blank corrected, thereafter normalised to internal standards and host dry weight, and analysed using MetaboAnalyst 6.0 (Pang et al. 2024). The data were log transformed and their normality and homogeneity were visually confirmed. First, principal component analyses (PCA) were conducted using all samples and metabolites, to assess the effect of temperatures (ambient and elevated), anemone groups (SS8, SS9 and WT10) and sampling days (Day 31 and 36). Then, the overall temperature effect was evaluated (regardless of anemone groups, Day 31 or Day 36). Metabolites that were significantly different between ambient and elevated temperatures were identified with *t*‐test and fold change analysis, and thereafter visualised with a heatmap using Euclidean distance and the Ward clustering algorithm. A metabolite was considered significant when *p*_adj_ < 0.05 and when fold change > 30%. Metabolites that were significantly different were then manually matched to the *Acropora digitifera* KEGG pathway map (a scleractinian in the same class (Anthozoa) as *E. diaphana* ) and visualised. Note that only pathways with at least three metabolite matches were shown. Next, the effect of temperature within specific anemone groups was examined. The data were subset into specific anemone groups (SS8‐, SS9‐ or WT10‐anemones) and tested for the effect of temperature within a group as described earlier (i.e., PCA, *t*‐test, fold change analysis and pathway match). Finally, the effect of anemone group within a treatment was examined. The data were subset into ambient or elevated treatment, where the effect of anemone groups was tested with an ANOVA. Metabolites with significant differences in abundance were visualised as a heatmap.

## 3 Results

### 3.1 Symbiodiniaceae Identity

ITS2 metabarcoding confirmed that the *E. diaphana* in this study had maintained the same ITS2 profile over the 1.5 years of culturing since inoculation (Tsang Min Ching et al. 2022) (Figure S2). No cross‐contamination or reappearance of the homologous *B. minutum* was observed in anemones hosting the various *C. proliferum* strains.

### 3.2 Photochemical Efficiency (Fv/Fm)

All anemone groups showed relatively stable photochemical efficiency throughout the experiment under ambient temperature, but a reduction was observed under elevated temperature for all groups (Figure 2). The day when a significant Fv/Fm reduction under elevated temperature (compared to ambient) was first detected differed between anemone groups (Figure 2, Tables 1 and S2). Under an elevated temperature of 32°C, the first group to show a significant decline in photochemical efficiency were the SS9‐anemones (Day 12), followed by SS1‐ and SS5‐anemones (Day 18), and SS7‐ and SS3‐anemones (Day 26) (Figure 2, Tables 1 and S2). After Day 26, the temperature of the elevated treatment was increased to 33°C. The next anemone group to show a significant drop in photochemical efficiency were the WT10‐anemones (Day 28), followed by B1‐ and SS8‐anemones (Day 30) (Figure 2, Tables 1 and S2). For all anemone groups, the significant difference in photochemical efficiency between ambient and elevated temperature continued to all later time points, after the first day it was detected.

**TABLE 1:** The first day when photochemical efficiency was significantly lower under elevated temperature than ambient temperature in each anemone group. The decline continued to be significant from the first significant day until the end of the experiment.

Anemone group	First significant day	Temperature	Thermotolerance rank
B1, SS8	Day 30	33°C	1
WT10	Day 28	33°C	2
SS7, SS3	Day 26	32°C	3
SS1, SS5	Day 18	32°C	4
SS9	Day 12	32°C	5

### 3.3 Symbiodiniaceae Cell Density

**FIGURE 3:**
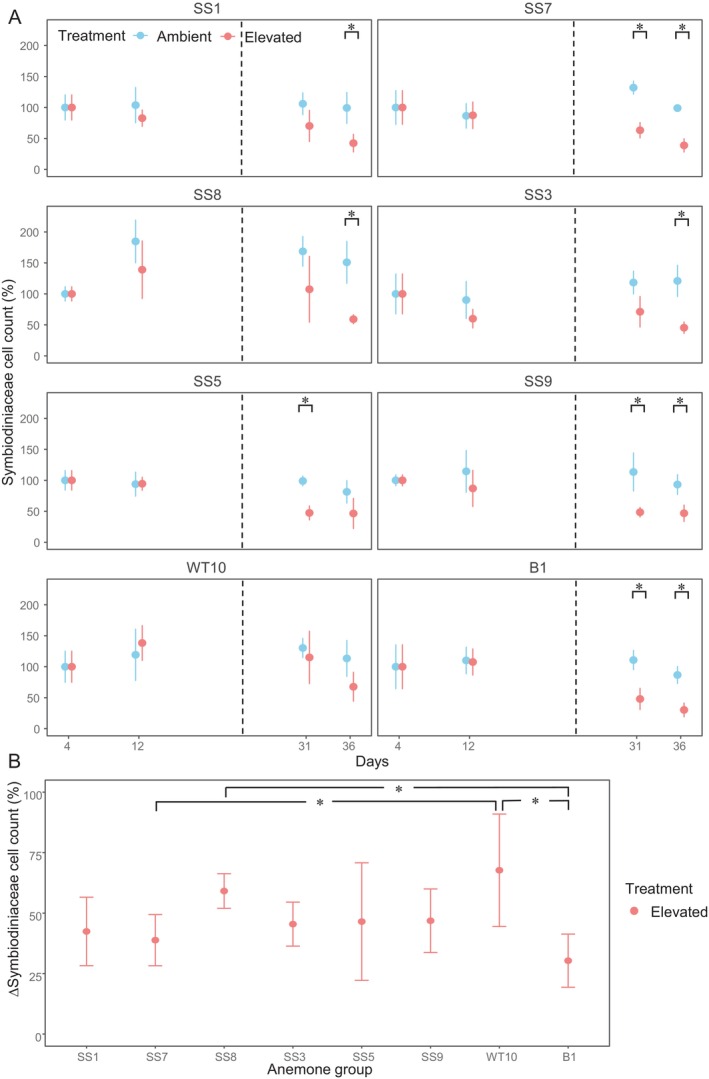
Symbiodiniaceae cell density of the anemone groups. Percentage change in Symbiodiniaceae cell counts (A) throughout the experiment, relative to the Day 4 (temperature effect), (B) on Day 36 under elevated temperature treatment, relative to Day 4 (anemone group effect). * indicates significant differences between temperatures (A) or anemone group under elevated temperature (B) (Tables S3 and S4). Biological replication was *n* = 4 per anemone group per temperature per day. The dash line indicates when the elevated temperature treatment reached 33°C.

Based on the Symbiodiniaceae cell counts normalised to host protein content, B1‐anemones had approximately double the amount of Symbiodiniaceae cells compared with all anemone groups hosting *C. proliferum* at the beginning of the experiment (Day 4) (Figure S3). Under ambient temperature, all anemone groups showed a relatively stable Symbiodiniaceae cell density throughout the experiment (Figures 3A and S3). Except for WT10‐anemones, a significant reduction in Symbiodiniaceae cell density was detected in all anemone groups under elevated temperature, compared with ambient temperature (Figure 3A). The reductions were first observed in SS5‐, SS7‐, SS9‐ and B1‐anemones on Day 31, followed by SS1‐, SS3‐ and SS8‐anemones on Day 36 (Figure 3A, Table S3). By the end of the experiment (Day 36), the relative cell density (relative to Day 4) of WT10‐anemones (67.7% ± 23.2%) and SS8‐anemones (59.1% ± 7.2%) was higher than for B1 (30.3% ± 11.0%); and the WT10‐anemone group also had a higher relative cell density than SS7 (38.8% ± 10.6%) (Figure 3B, Table S4). For the above reasons, the WT10‐anemone group was ranked as the most thermally tolerant group for this trait. The other anemones were ranked, based on the thermotolerance from highest to lowest; SS8‐anemones > SS1‐ and SS3‐ anemones > SS5‐ and SS9‐anemones > SS7‐anemones and > B1‐anemones (Table 2).

**TABLE 2:** Summary of anemone group thermotolerance ranking based on the various physiological measurements. The lower the score, the higher the thermotolerance of that anemone group.

Strain	Species	Category	Photochemical efficiency	Symbiodiniaceae cell counts	Photosynthesis	Total score
SS8^a^	*Cladocopium proliferum*	Heat‐evolved, conferring	1	2	1	4
WT10^a^	*Cladocopium proliferum*	Wild‐type	2	1	2	5
SS1	*Cladocopium proliferum*	Heat‐evolved, conferring	4	3	1	8
B1	*Breviolum minutum*	Homologous	1	6	1	8
SS3	*Cladocopium proliferum*	Heat‐evolved, non‐conferring	3	3	NA	6^b^
SS5	*Cladocopium proliferum*	Heat‐evolved, non‐conferring	4	4	NA	8^b^
SS7	*Cladocopium proliferum*	Heat‐evolved, conferring	3	5	NA	8^b^
SS9^a^	*Cladocopium proliferum*	Heat‐evolved, non‐conferring	5	4	NA	9^b^

*Note:* The total score was calculated by adding up the scores across the three measured traits. NA indicates that no measurement was taken.

^a^ Anemone groups selected for host metabolome study.

^b^ No measurement was taken for photosynthesis for these anemone groups. Hence the total score cannot be compared directly with groups for which this trait was measured.

### 3.4 Gross Photosynthesis and Respiration Rate

Under ambient temperature, the gross photosynthesis rate did not differ between anemone groups (Figure S4). Under elevated temperature, WT10‐anemones were the first group to show a significantly lower gross photosynthesis rate by Day 12. By Days 31 and 36, all measured anemone groups (SS1‐, SS8‐, WT10‐, B1‐anemones) had a significantly lower gross photosynthesis rate at 33°C compared with ambient temperature (27°C) (Figure S4, Table S5). For the respiration rate, there was no significant difference between temperatures and anemone groups throughout the measurement days (Figure S5, Table S5).

### 3.5 Summary of Physiological Measurements

Table 2 summarises the physiological measurements and the thermotolerance ranking of the anemone groups. The lower the total score, the higher the thermotolerance of an anemone group. Note that photosynthesis rate was only measured in four anemone groups, therefore, the total score cannot be directly compared with anemone groups that were not measured. Based on the total score, the SS8‐anemones represent the most thermally tolerant group, followed by WT10‐anemones. The SS9‐anemones were most thermally sensitive (note that photosynthesis rate was not measured). These three anemone groups were therefore selected for the host metabolome study.

### 3.6 Anemone Host Metabolome

#### 3.6.1 Between Temperature Treatments

**FIGURE 4:**
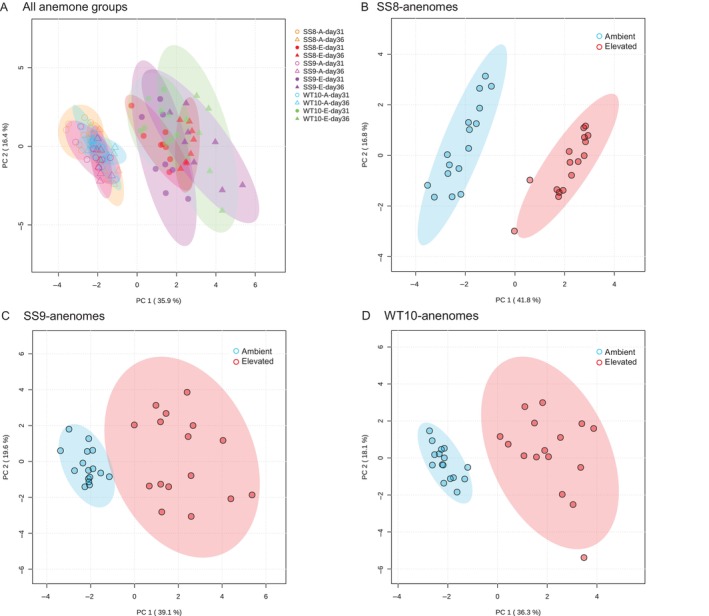
PCA plots of the *E. diaphana* host using all 184 metabolites. (A) All anemone groups, temperatures and days. Data points were coloured per anemone group per treatment (*n* = 8). Circle and triangle points represent Day 31 and 36, and unfilled and filled shapes represent ambient and elevated temperatures, respectively. (B) SS8‐ (C) SS9‐ and (D) WT10‐anemones between temperatures, Day 31 and 36 combined. A = ambient temperature, E = elevated temperature.

**FIGURE 5:**
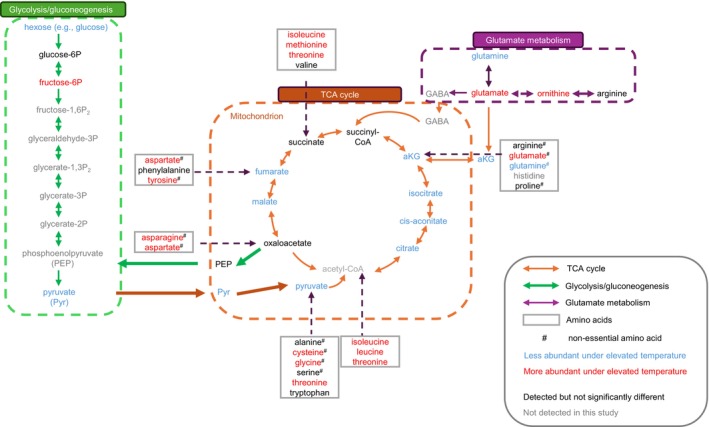
Summary of *E. diaphana* host metabolome under ambient versus elevated temperature, focusing on the TCA cycle, glycolysis/gluconeogenesis and amino acids. The entry points of amino acids into the TCA cycle are shown, note that some amino acids can enter from multiple entry points. aKG refers to alpha ketoglutarate.

A total of 184 metabolites were detected in the host fraction of the three anemone groups (SS8‐, SS9‐ and WT10‐anemones) by LCMS (Chan et al. 2024; Appendix data S1). PCA using all samples and metabolites showed that temperature was the main driver of the grouping (Figure 4A), with 117 metabolites being significantly different between ambient and elevated temperature (Figure S6, Table S6), covering multiple categories (Table 3). Elevated temperature consistently resulted in increased levels of most detected amino acids (10 out of 19), while levels of key intermediates in central carbon metabolism, including pyruvate and all detected intermediates in the tricarboxylic acid (TCA) cycle (including malate, fumarate, citrate, cis‐aconitate, isocitrate, α‐ketoglutarate) were consistently decreased following exposure to elevated temperature (Figure 5). Variable changes were detected in the levels of different neutral sugars, sugar phosphates and disaccharides. For example, while levels of glucosamine, sugar alcohols, some hexose phosphates (fructose 6‐phosphate, glucose 1‐phosphate), rhamnose and one of the pentose species increased in heat stressed anemones; levels of glucosamine‐6‐phosphate, gluconic acid, hexose, one of the pentose species and several disaccharide species decreased (Figure 5, Tables 3 and S6–S9).

**TABLE 3:** Pathways and fold change (FC) of metabolites that were significantly different between temperatures.

Category	Metabolite	A vs. E	SS8 (A vs. E)	SS9 (A vs. E)	WT10 (A vs. E)
Amino acid	L‐asparagine	0.64	0.67	0.63	0.62
	L‐glutamine	1.52	1	1.53	1.89
	L‐cysteine	0.49	0.35	0.55	0.61
	Glycine	0.5	0.47	0.45	0.62
	L‐aspartate	0.64	0.67	0.63	0.62
	L‐methionine	0.6	0.64	0.6	0.6
	L‐lysine	0.62	0.58	1	1
	L‐isoleucine	0.75	0.74	0.7	1
	L‐threonine	0.55	0.54	1	1
	L‐tyrosine	0.27	0.26	0.21	0.35
	L‐glutamate	0.57	0.55	0.55	0.61
	L‐alanine	1	0.74	1	1
Carbohydrate metabolism	D‐glucose 1‐phosphate	0.73	1	0.72	0.67
	D‐fructose 6‐phosphate	0.73	1	0.71	0.67
	D‐glucosamine	0.58	1	1	1
	D‐glucosamine 6‐phosphate	1.91	1.91	1.8	2.05
	N‐acetyl‐D‐glucosamine	0.65	0.75	0.6	0.6
Citrate cycle (TCA cycle)	(S)‐malate	1.49	1.44	1.7	1
	Citrate	1.91	2.19	2.52	1.33
	Fumarate	1.42	1.35	1.64	1
	cis‐Aconitate	2.77	2.71	2.91	2.69
	Isocitrate	3.24	3.09	4.4	2.61
	2‐oxoglutarate	2.06	2.13	1.92	2.16
	Pyruvate	1.53	1.47	1.74	1.37
Glycolysis/gluconeogenesis	D‐glucose 1‐phosphate	0.73	1	0.72	0.67
	D‐glucose 6‐phosphate	1	1	1	0.71
	Hexose sugar	3.35	3.28	4.6	2.56
	Pyruvate	1.53	1.47	1.74	1.37
Disaccharide, pentose sugar, sugar alcohol	Disaccharide_a	6.13	4.84	6.35	7.55
	Disaccharide_c	6.01	4.57	6.26	7.61
	Disaccharide_d	1	1	1	0.24
	Pentose sugar_a	2.62	2.47	2.98	2.38
	Pentose sugar_b	0.59	0.62	0.59	0.57
	Sugar alcohol_a	0.27	0.26	0.21	0.35
	Sugar alcohol_b	0.27	0.26	0.21	0.35
	Sugar alcohol_c	0.27	0.26	0.21	0.35
Purine metabolism	D‐ribose 5‐phosphate	0.49	0.49	0.46	1
	IMP	0.44	1	0.38	0.35
	Xanthine	0.71	1	0.62	0.75
	Guanine	0.59	0.66	0.46	0.67
	dGMP	2.3	2.37	2.64	2.01
	GMP	0.63	1	0.62	0.54
	dAMP	3	3.04	2.97	3
	Adenosine	ns	1	1	1.65
Pyrimidine metabolism	L‐glutamine	1.52	1	1.53	1.89
	Uridine	0.68	0.61	0.63	1
	UMP	0.41	0.46	0.37	0.4
	CMP	0.6	0.45	1	1
	dCMP	1.97	2.1	1	1.98

*Note:* Red font: FC of metabolites that were significantly more abundant in elevated samples (e.g., FC 0.5 = 2 times more abundant under elevated temperature). Blue font: Metabolites that were significantly less abundant in elevated samples (e.g., FC 2 = 2 times less abundant under elevated temperature). A FC of 1 suggests that the metabolite that was not significantly different between temperatures. A = ambient treatment, E = elevated treatment. Only pathways with at least three matches are shown. Biological replicate was *n* = 16 (Day 31 and 36 combined) per anemone group per temperature.

#### 3.6.2 Temperature Effect Within Anemone Groups

Next, the effect of temperature treatment within an anemone group was investigated. PCA within the same anemone group showed clear separation between temperature treatments for all groups (Figures 4B–D and S7), but limited separation between Day 31 and 36 within the same temperature (Figure S7). For this reason, subsequent analyses focusing on temperature effect combined data from Day 31 and 36. Between temperatures (Day 31 and 36 combined), there were 107, 114 and 94 significant metabolites in SS8‐, SS9‐ and WT10‐anemones, respectively (Tables S7–S9), covering multiple categories (Table 3). Consistent with the overall ambient versus elevated comparison, all anemone groups under elevated temperature showed an increase in amino acid abundance, including amino acids required for glutathione synthesis (i.e., cysteine, glutamate and glycine), and a marked decrease in TCA metabolites, compared to their ambient counterparts (Table 3). Nevertheless, the extent of difference (fold change) between temperature noticeably differed among anemone groups, particularly for non‐amino acid and TCA cycle intermediates (Tables 3 and S7–S9). The reduction in hexose (e.g., glucose) was the greatest in SS9‐anemones (4.6‐fold) and the reduction of disaccharides (e.g., cellobiose, trehalose) was the greatest in WT10‐anemones (7.6‐fold) under a simulated heatwave. In contrast, SS8‐anemones had lower reduction in both hexose (3.3‐fold) and disaccharides (4.6–4.8‐fold). The greatest increase in glutathione precursors and sugar alcohols (e.g., galactitol, mannitol, sorbitol) under elevated temperature occurred in the SS8‐anemone group, whereas WT10‐anemones experienced the least increase (Table 3, S7–S9).

#### 3.6.3 Anemone Group Effect Within Temperature Treatment

**FIGURE 6:**
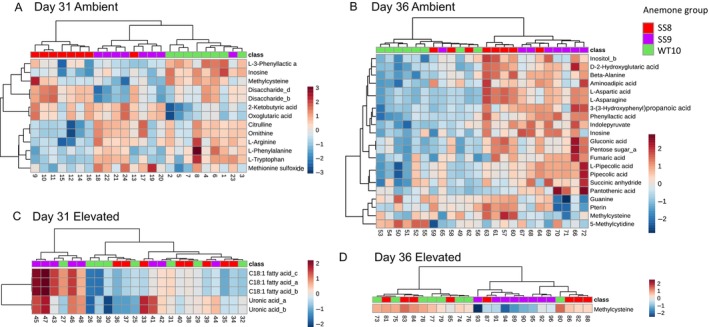
Heatmap of metabolites that were significantly different between *E. diaphana* hosts in the same temperature treatment and on the same sampling day. (A) Ambient treatment Day 31, (B) ambient treatment Day 36, (C) elevated treatment Day 31, (D) elevated treatment Day 36. Sample and metabolite similarities were calculated based on Euclidean distance and the Ward clustering algorithm. A = ambient temperature, E = elevated temperature.

Within the same temperature treatment, anemone groups showed more significant differences under ambient than elevated temperature. Under ambient temperature, 13 and 21 metabolites were significantly different among anemone groups on Day 31 and 36, respectively (ANOVA, Figures 6A,B and S8, Tables S10–S11). Under elevated temperature, only five metabolites were significantly different among anemone groups (ANOVA: C18:1 fatty acids galacturonic acid and uronic acid_a) on Day 31, all of which were significantly higher in SS9‐anemones (Figure 6C, Table S12). By Day 36, only one metabolite was significantly different among anemone groups under elevated temperature (ANOVA; methylcysteine) (Figure 6D, Table S12).

## 4 Discussion

### 4.1 Heterologous *C. proliferum* Maintained a Long‐Term, Healthy Symbiosis With *E. diaphana*

ITS2 metabarcoding results showed that *E. diaphana* can maintain a symbiosis with the heterologous *C. proliferum* for at least 1.5 years, demonstrating that *E. diaphana* is a valid model for studying long‐term cnidarian‐algal symbiosis. Prior to this study, there has only been one other report showing long‐term symbiosis (i.e., > 1 year) between *E. diaphana* inoculated with heterologous Symbiodiniaceae (Chen et al. 2016). While the homologous *B. minutum* had an ~2‐fold higher Symbiodiniaceae cell density than all heterologous *C. proliferum* strains under ambient temperature, this is not unexpected as cell equilibrium densities in cnidarians differ between symbiodiniacean genera in nature (Turnham et al. 2023). Heterologous Symbiodiniaceae are often considered suboptimal to cnidarian hosts relative to homologous species. Examples of negative effects include reduced photosynthesis rates and host growth (Gabay, Weis, and Davy 2018), reduced symbiont carbon assimilation (Sproles et al. 2019), increased host respiration (Starzak et al. 2014), as well as increased host innate immunity responses and lipid catabolism (Matthews et al. 2017).

Based on our physiological data (photochemical efficiency, Symbiodiniaceae cell count, photosynthesis and respiration) and the fact that heterologous *C. proliferum* maintained a long‐term functional symbiosis with anemones, heterologous *C. proliferum* was likely an equally beneficial symbiont to the anemone host as homologous *B. minutum* . Under ambient temperature, photosynthesis and respiration rates did not differ between anemone groups. Under elevated temperature, anemones hosting the heterologous SS8 and WT10 had higher thermotolerance rankings based on a number of physiological traits compared with those hosting homologous B1 (Table 2). Since the *E. diaphana* and heterologous *C. proliferum* were both from the GBR in this study, these symbionts could be more compatible and beneficial to the host than heterologous symbionts from different parts of the world (e.g., Gabay, Weis, and Davy 2018; Sproles et al. 2019). Future studies should further investigate the nutritional budget of these symbioses with heterologous symbionts and test if they translocate a similar amount of photosynthate to the host.

### 4.2 Symbiodiniaceae Strain Determined *E. diaphana* Thermal Tolerance

Our data suggest that the thermotolerance of *E. diaphana* is strongly influenced by the *C. proliferum* strain it harbours (Table 2). Based on the combined ranking from physiological data (photochemical efficiency, Symbiodiniaceae cell density, gross photosynthesis), SS8‐anemones represented the most thermotolerant group. Consistent with this observation, SS8 also confers high bleaching tolerance to coral larvae (Buerger et al. 2020), juveniles (Quigley et al. 2023) and adults (Chan et al. 2023) compared with WT10, indicating that this strain will likely enhance thermotolerance in other cnidarian host species and life stages. In contrast, SS5‐ and SS9‐anemones, as well as the homologous B1‐anemones exhibited limited thermotolerance. This is also consistent with a previous study (Buerger et al. 2020), where SS5 and SS9 did not confer enhanced bleaching tolerance to coral larvae. However, other heat‐evolved strains that previously conferred thermotolerance to corals (SS1 and SS7) (Buerger et al. 2020; Quigley and van Oppen 2022; Quigley et al. 2023) did not confer high thermotolerance to the *E. diaphana* in this study. In contrast, the WT10 strain, which has not been through experimental evolution, conferred the second highest level of thermotolerance on its host. The WT10‐anenome was ranked second after SS8‐anenomes because it experienced an earlier onset of gross photosynthesis decline under elevated temperature.

Since high Symbiodiniaceae density increases the susceptibility of corals to bleaching (Cunning and Baker 2013), anemones with higher pre‐stress cell densities were expected to experience more severe bleaching. The cell density and thermotolerant differences between B1‐anemones (hosting *B. minutum* ), and WT10‐ and SS8‐anemones (hosting *C. proliferum* ) could be a consequence of host‐symbiont specificity (Gabay et al. 2019). B1‐anemones had the highest Symbiodiniaceae cell density among all anemone groups prior to a simulated summer heatwave (Day 4, Figure S3) and exhibited the greatest decline in symbiont cell density under heat stress. Additionally, B1‐anemones maintained higher photochemical efficiency and gross photosynthesis rates to later time points under heat stress. This combination of high cell density, photochemical efficiency and photosynthesis under high temperature in B1‐anemones may have led to excessive ROS production by Symbiodiniaceae. This likely triggered a cellular cascade within the anemone holobiont, resulting in the loss of the Symbiodiniaceae from the host cells, which corresponds to its greatest decline in symbiont cell density among all anemone groups. In line with this hypothesis, WT10‐ and SS8‐anenomes, which had lower pre‐stress cell densities than B1‐anemones, experienced less bleaching as expected (Table 2). For WT10‐anemones, their earlier reduction in photosynthesis rates than other anemone groups may have resulted in lower ROS production under heat stress, and hence may explain their reduced symbiont loss. Alternatively, lower photosynthesis rates may reflect physiological stress in WT10 under elevated temperatures.

Nonetheless, high holobiont thermotolerance in WT10‐anemones was unexpected. The high Symbiodiniaceae cell density of WT10‐anemones under elevated temperature was not an artefact of protein normalisation, as WT10‐anemones did not have particularly lower protein content than other *C. proliferum* groups (see Symbiodiniaceae cell density raw data, Table S13). This contrasting result may be explained by the different host organism used here (anemones instead of corals) and the possible loss of thermotolerance in certain heat‐evolved Symbiodiniaceae strains over time. While sea anemones (e.g., *E. diaphana* ) have been a popular coral model because of their fast growth rate and easy maintenance (Weis et al. 2008), a recent study showed that their oxidative stress response under elevated temperature differed significantly with that of corals, challenging their suitability as a model to study coral bleaching cellular mechanisms (Doering, Maire, et al. 2023).

Additionally, the anemones of this study were kept under ambient temperature (27°C) for 1.5 years prior to heat exposure. As somatic mutations in the symbiont genomes continue to occur within the anemone host (albeit slower than in culture given the slower growth rates of the symbiont in hospite relative to in vitro), mutations that provide benefits under ambient temperature may have arisen (van Oppen et al. 2011). Some heat‐evolved strains may have lost mutations that are beneficial under high temperature but detrimental or neutral under ambient conditions. In this context, the cryopreservation of heat‐evolved Symbiodiniaceae may be critical if this approach is to be scaled up for reef restoration. Nevertheless, corals inoculated with heat‐evolved SS8 and maintained under ambient temperature for ~5–10 months still showed enhanced thermotolerance compared to corals inoculated with WT10 (Chan et al. 2023; Quigley et al. 2023), suggesting that at least some strains maintained beneficial mutations under medium‐ to long‐term ambient conditions.

### 4.3 Heat Stress Triggered Similar Host Metabolomic Responses, but the Extent Differed Between Symbiodiniaceae Strains Harboured

Despite differences in physiological performance, the major metabolic responses of the host across the most (SS8‐, WT10‐anemones) and least (SS9‐anemones) thermally tolerant anemone groups under elevated temperatures were similar. Significantly, elevated temperatures led to significant increases or decreases in the majority of detected metabolites, highlighting the substantial rewiring of anemone metabolism at elevated temperatures and/or as a consequence of the specific Symbiodiniaceae‐ *E. diaphana* pairing. As the heat stress progressed from Day 31 to 36, fewer metabolites became significantly different among anemone groups (from five to one), indicating that heat stress generated a ‘universal’ response in anemones, regardless of the Symbiodiniaceae species or strain they harboured. It is also possible that the influence of Symbiodiniaceae strain to host metabolome reduced as their cell density progressively declined under elevated temperature, leaving a common host heat stress response as the dominant pattern detected. Major responses included an increase in amino acids (including precursors of glutathione synthesis), and concomitant decrease in TCA intermediates and products. Nevertheless, the extent of difference in these responses between ambient and elevated temperatures was noticeably different among anemone groups, suggesting that Symbiodiniaceae strains can influence host metabolome and nutritional budget.

#### 4.3.1 Increased Glutathione Metabolism and Abundance of Amino Acids

Under elevated temperature, all anemone groups showed an increase in metabolites that are part of glutathione metabolism, which plays a key role in cellular defence against oxidative stress (Diaz‐Vivancos et al. 2015). The tripeptide glutathione is composed of glutamate with a gamma peptide bond to glycine and cysteine; it reacts with ROS to prevent oxidative damage to other biomolecules. In this study, a higher abundance of glutathione precursors, glutamate, glycine and cystine was found under elevated temperature, which may have facilitated the synthesis of glutathione. However, glutathione itself was not detected in our samples. Nevertheless, Hillyer et al. (2016) detected an increase in glutathione abundance in *E. diaphana* tissues under high temperatures. In this study, sugar alcohols such as mannitol and sorbitol were more abundant in all anemone groups under elevated temperature as well. These polyols are also antioxidant compounds in cnidarians (Lesser 1997; Dungan, Maire, et al. 2022) and plants (Patel and Williamson 2016; Li et al. 2023), raising the possibility that the accumulation of antioxidant metabolites (glutathione, polyols) is a common anemone host response to heat stress, and supporting the likely role of ROS in coral bleaching. The greatest fold change in glutathione precursors and sugar alcohols between temperatures occurred in SS8‐anemones, whereas WT10‐anemones experienced the lowest fold change. It is possible that experimental evolution has increased the ability of heat‐evolved strains to produce and translocate antioxidant compounds, resulting in these algal symbiont‐derived compounds to be measured in the host fraction. To verify this, further study is needed to focus on the detection of precursors as well as the final products of antioxidant compounds (e.g., glutathione, ubiquinone, dimethylsulphide, dimethylsulphoniopropionate, zeaxanthin, ascorbic acid) in anemone groups (Lesser 2006; Doering, Tandon, et al. 2023; Maire et al. 2023). When clonal individuals are used as the hosts, as was done in our study, differences found in antioxidant compound abundance in the hosts carrying different Symbiodiniaceae strains can be attributed to symbiont contribution.

In this study, all anemone groups experienced an increase in amino acid abundance under elevated temperature. Such an increase has been observed in this anemone previously (Hillyer et al. 2016), although it did not occur in as many amino acid species as in our study. The increase in amino acid could either reflect global decreases in the rate of protein synthesis and/or increased autophagy. Autophagy mediates the non‐specific degradation of cytoplasmic proteins, as well as various organelles and is commonly upregulated in response to stresses such as nutrient and energy deprivation (He and Klionsky 2009; Parzych and Klionsky 2014). Amino acids generated by autophagy can be used to produce energy via the TCA cycle and oxidative phosphorylation (e.g., oxaloacetate, fumarate), sugar phosphate via gluconeogenesis (Figure 5) and to generate new stress response proteins.

As the anemone metabolome of this study was analysed at a steady state (i.e., without studying metabolic fluxes), one cannot distinguish whether the increase in amino acids was due to an increase in autophagy, increased de novo synthesis or reduced utilisation (e.g., for protein synthesis). Studies have found increased proteolysis (Petrou et al. 2021), upregulation of amino acid catabolic degradation pathways and downregulation of amino acid biosynthetic pathways (Rädecker et al. 2021) in coral hosts *Acropora millepora* and *Stylophora pistillata* under heat stress. Considering that all anemone groups exhibited signs of heat stress (i.e., reduced photochemical efficiency, Symbiodiniaceae cell density), increased autophagy is more likely to have occurred rather than increased *de novo* synthesis.

#### 4.3.2 Reduced TCA Metabolites and Some Sugar Species

Elevated temperature resulted in a decrease in the levels of all intermediates in the TCA cycle in all anemone hosts, suggesting general repression of mitochondrial metabolism. The greatest fold change between temperatures (i.e., lowest abundance under elevated temperature) occurred in SS9‐anemones, the least thermotolerant group. A reduction in some TCA intermediates has previously been observed in Symbiodiniaceae under heat stress (Hillyer, Diaz, Lutz, Wilkinson, et al. 2017; Haydon et al. 2023; Matthews et al. 2024), although similar changes were not found in the coral host *Pocillopora acuta* (Haydon et al. 2023) or *Acropora aspera* (Hillyer, Diaz, Lutz, Wilkinson, et al. 2017). The mitochondrial TCA cycle drives oxidative phosphorylation, a major source of ATP synthesis in most animal cells. Increased temperature can lead to increased leakage of electrons from different respiratory complexes in the electron transport chain, and production of toxic ROS (primarilyO_2_). The downregulation of TCA cycle fluxes in heat‐treated anemones could therefore reflect a protective response to reduce oxidative stress. Similarly, the concomitant decrease in intracellular pools of disaccharides could reflect increased ATP synthesis via glycolysis, which may be needed to compensate for loss of mitochondrial oxidative phosphorylation. Interestingly, a gene expression study has shown the downregulation of the TCA cycle in the coral *S. pistillata* under thermal stress (Rädecker et al. 2021), suggesting that this may be a general strategy in other host‐symbiont systems.

The TCA cycle intermediates also support many anabolic pathways, such as amino acid, nucleotide and fatty acid biosynthesis (reviewed in Martínez‐Reyes and Chandel 2020). For example, citrate can be exported to the cytosol and converted into oxaloacetate and acetyl‐CoA by citrate lyase to facilitate nucleotide and lipid synthesis, respectively. Decreased flux through the TCA cycle during heat stress could therefore also reflect a general decrease in growth rate and/or a shift to a metabolically quiescent state. As noted above, detailed dissection of TCA cycle fluxes will be needed to support these conclusions.

Under elevated temperatures, the level and direction of changes detected in different neutral sugars, sugar phosphates and disaccharides varied. Anemone groups showed a reduction in some sugars and disaccharides, which are likely to be photosynthates translocated to the host by Symbiodiniaceae (Burriesci, Raab, and Pringle 2012; Davy, Allemand, and Weis 2012; Matthews et al. 2018). However, some sugars and hexose phosphates increased instead under elevated temperatures. A decrease in glucose previously has been observed under high temperature in both cultured Symbiodiniaceae (Matthews et al. 2024) and coral holobionts (Williams et al. 2021), but an increase in galactose and glycerol has also been found in the host under heat stress (Hillyer, D. Dias, et al. 2017). In the host, a reduction in carbohydrates can be due to (1) a decline in gluconeogenesis in its associated Symbiodiniaceae; (2) a relationship shift from mutualism to parasitism, where Symbiodiniaceae retained more glucose for their own use (Yellowlees, Rees, and Leggat 2008; Lesser, Stat, and Gates 2013; Baker et al. 2018); or (3) increased oxidisation of glucose via glycolysis; or combination of the above. Dissection of these processes will require ^13^C‐tracer experiments, which we recommend being incorporated into future studies.

## 5 Conclusion

In summary, this study demonstrated that heterologous heat‐evolved *C. proliferum* can maintain long‐term symbiosis with *E. diaphana* and affect the thermotolerance, metabolome and nutritional budget of the host. Among the ten heat‐evolved strains, SS8 promoted higher holobiont thermotolerance compared to others. The host metabolome showed potential key mechanisms (i.e., autophagy, antioxidant, TCA cycle and gluconeogenesis/glycolysis) of *E. diaphana* heat stress responses, and the extent to which different algal symbionts influenced these responses. Future research will focus on measuring the underlying metabolic fluxes within and between the symbiont and host that explain these changes.

## Author Contributions

**Wing Yan Chan:** conceptualization, data curation, formal analysis, investigation, supervision, writing – review and editing, writing – original draft, methodology, project administration. **Rumi Sakamoto:** data curation, formal analysis, investigation, methodology, visualization, writing – original draft, writing – review and editing. **Talisa Doering:** writing – review and editing, data curation, formal analysis, methodology, investigation. **Vinod K. Narayana:** data curation, formal analysis, writing – review and editing, methodology, investigation. **David P. De Souza:** data curation, formal analysis, writing – review and editing, methodology, investigation. **Malcolm J. McConville:** formal analysis, supervision, investigation, writing – original draft, writing – review and editing, visualization, methodology. **Madeleine J. H. van Oppen:** conceptualization, funding acquisition, investigation, project administration, resources, supervision, writing – original draft, writing – review and editing.

## Conflicts of Interest

The authors declare no conflicts of interest.

## Supporting information

Supporting Information.

## Data Availability

All raw data and R codes for statistical analysis are available on Zenodo: https://doi.org/10.5281/zenodo.13824741 (Chan et al. 2024). Details of each file are provided in Table S13.
