# Supplementary material for: Heat‐Evolved Microalgae (Symbiodiniaceae) Are Stable Symbionts and Influence Thermal Tolerance of the Sea Anemone Exaiptasia diaphana
Source: Environ Microbiol. 2025 Jan 21;27(1):e70011. doi: 10.1111/1462-2920.70011 (PMC11751664; doi:10.1111/1462-2920.70011)
Supplement: Supplementary file 1 — Supporting Information. [file EMI-27-e70011-s001.docx]

APPENDIX

**Appendix data**

**Appendix Figures**

**
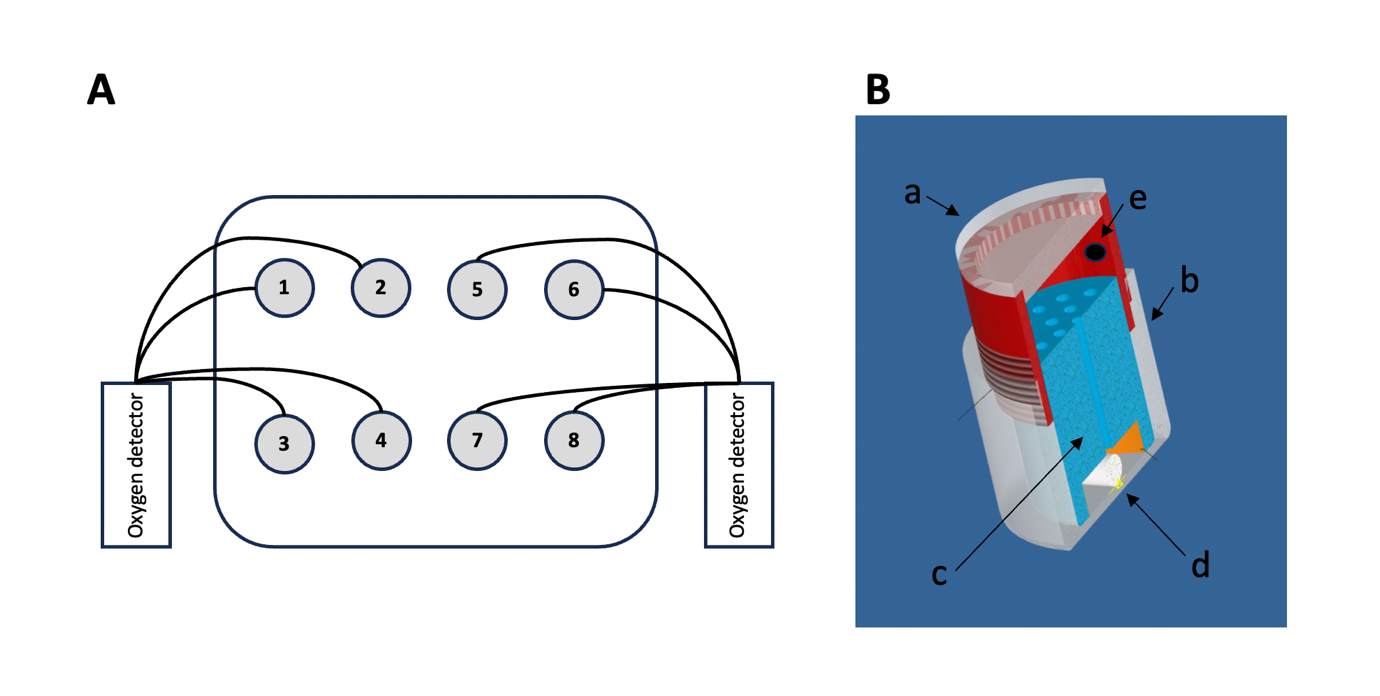
Figure S1. Respirometry setup.** (A) Two oxygen sensors, each attached to four sampling vials. (B) Each vial is designed to have a lid (a), base (b), stage (c) and a magnetic stirrer (d). Oxygen sensor (e) is attached on the lid of the vial. Image (B) in courtesy of the Australian Institute of Marine Science.


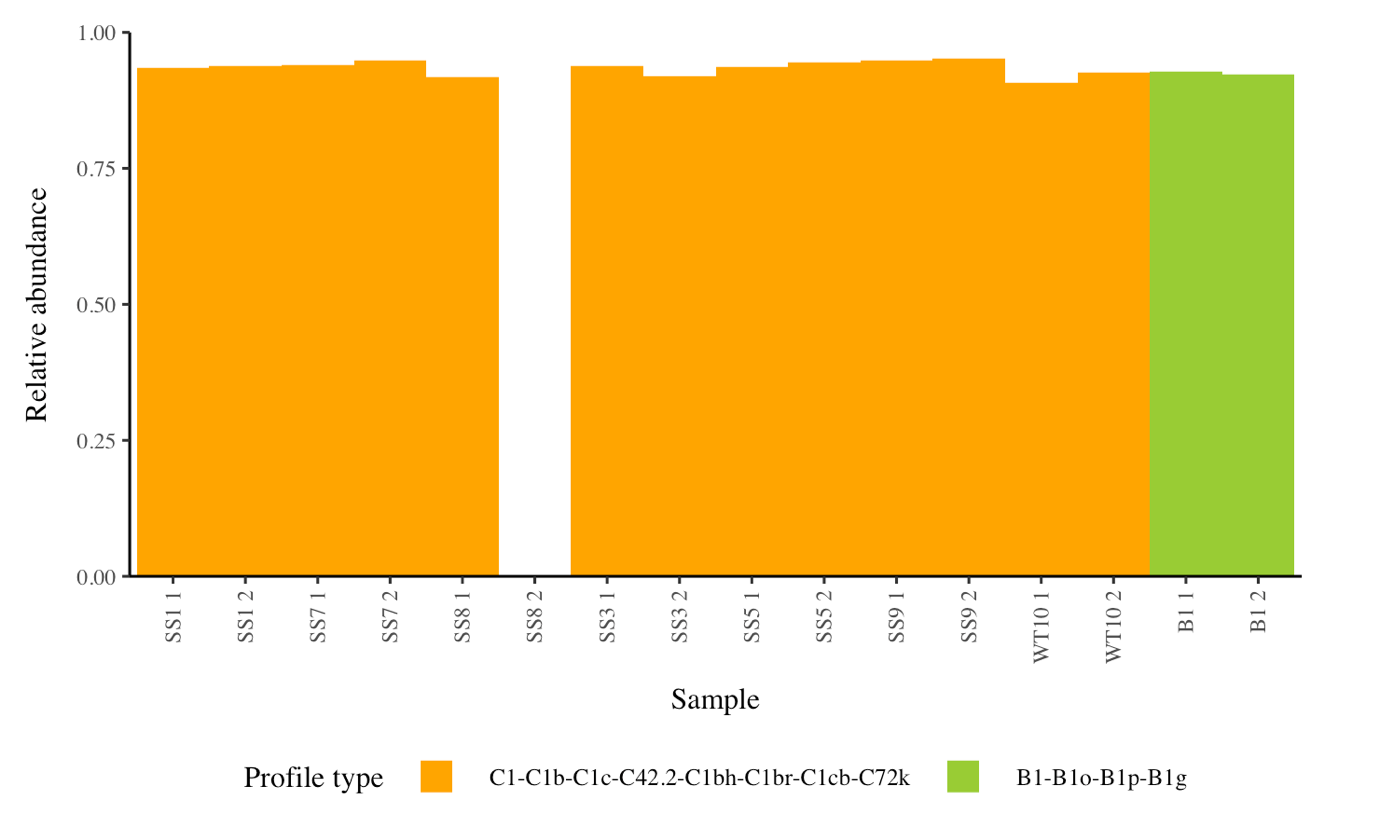


**Figure S2. ITS2 metabarcoding results.** *Breviolum minutum* (green) and *Cladocopium* *proliferum* (orange) were the only ITS2 profile types identified from the anemones used for the experiment. Note that ITS2 metabarcoding cannot distinguish between *C. proliferum* strains, but anemone groups were always grown in separate tanks. Biological replication was n = 2 per anemone group, the last number of the sample name indicate replicate 1 or 2. Sample SS8 2 did not amplify successfully and was excluded.


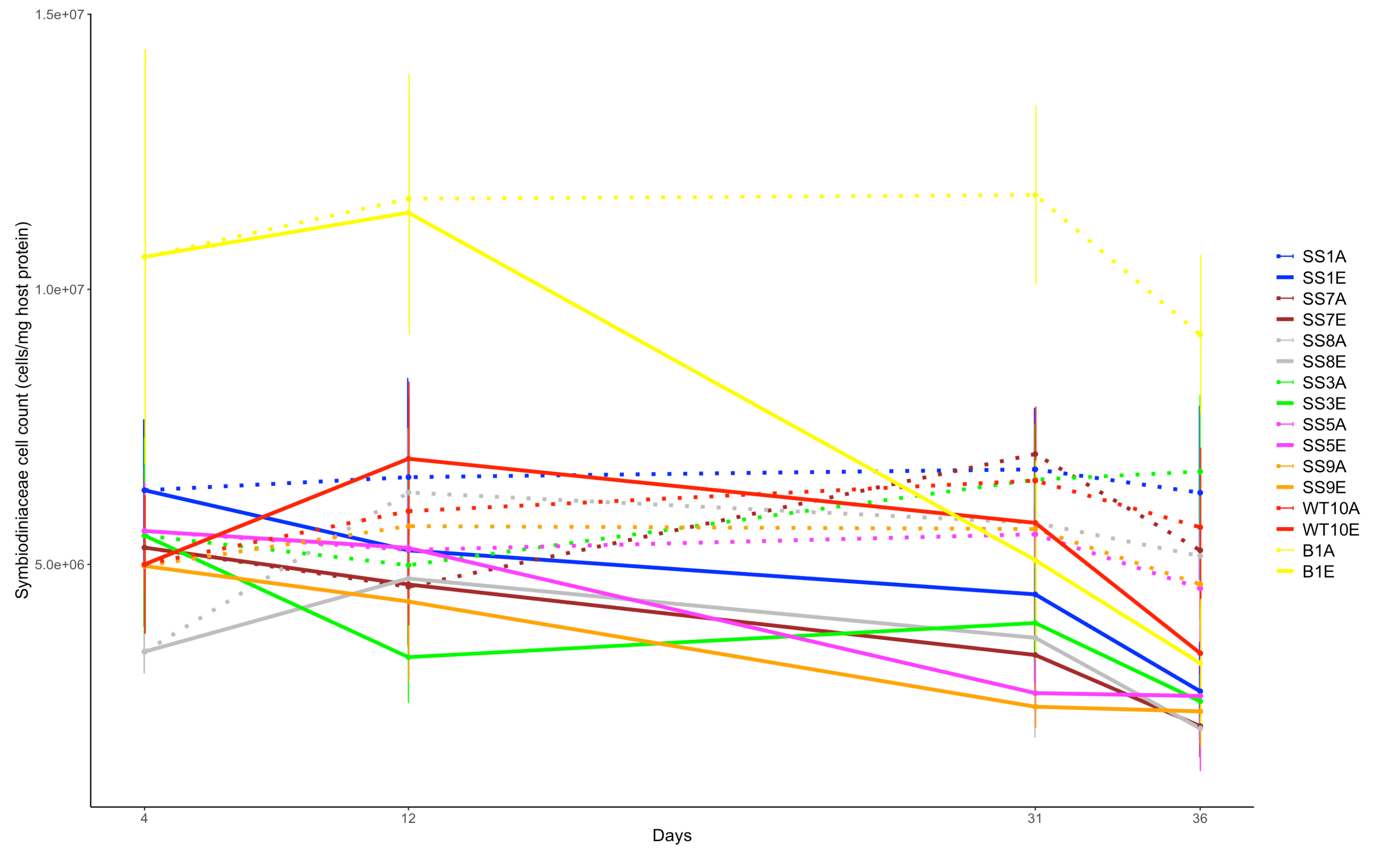


**Figure S3. Symbiodiniaceae cell count over time**, **normalized to host protein content.** A = ambient temperature, E = elevated temperature. Dotted lines indicate ambient and solid lines indicate elevated temperature treatments. Values are means ± 1SD. Biological replication was n = 4 per anemone group per temperature per day.

**
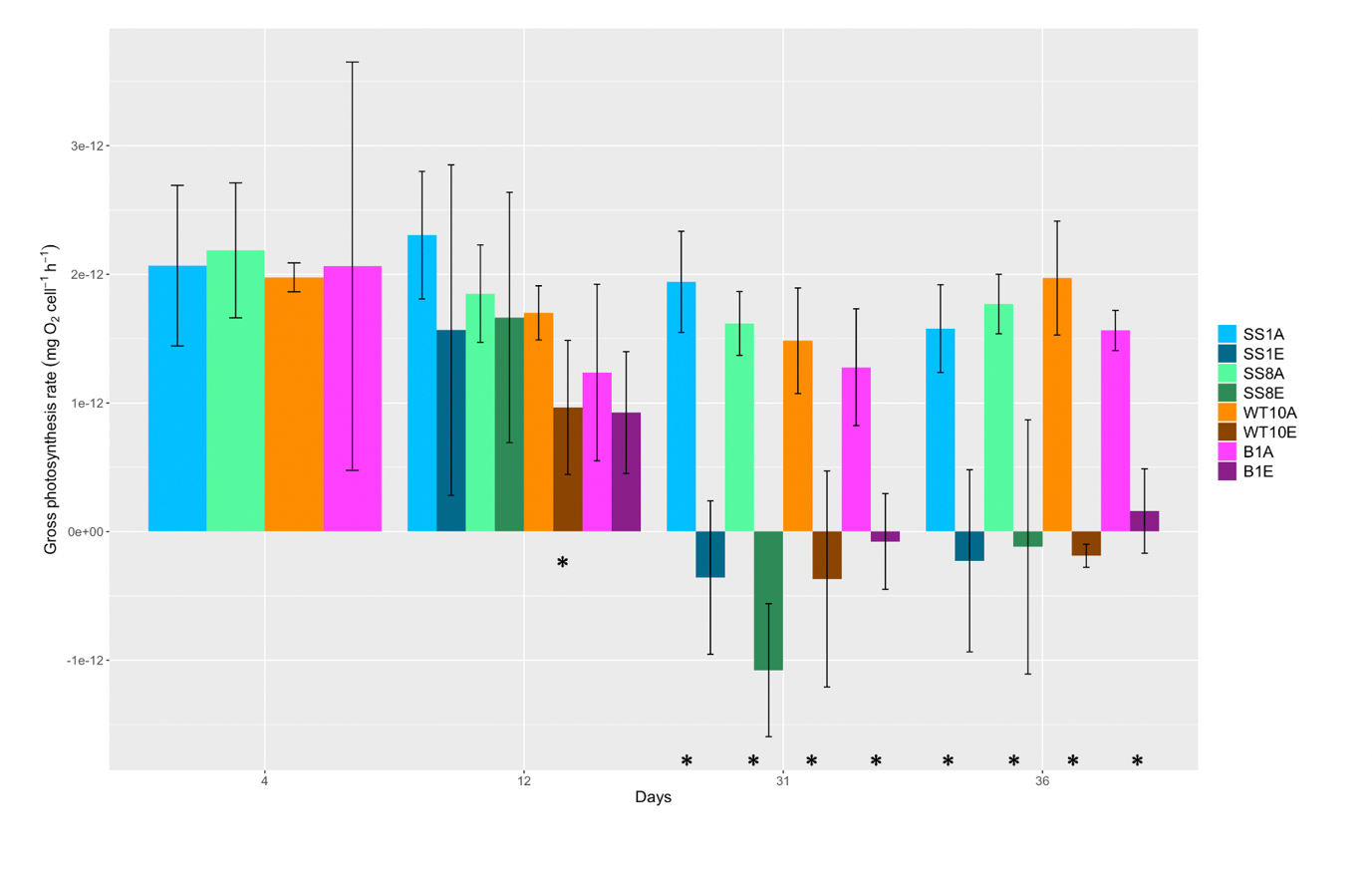
Figure S4. Gross photosynthesis rates of anemone groups** **normalized by Symbiodiniaceae cell count.** A = ambient temperature, E = elevated temperature. * indicates significant differences between temperature treatments. Values are means ± 1SD. Biological replication was n = 4 per anemone groups per temperature per day.


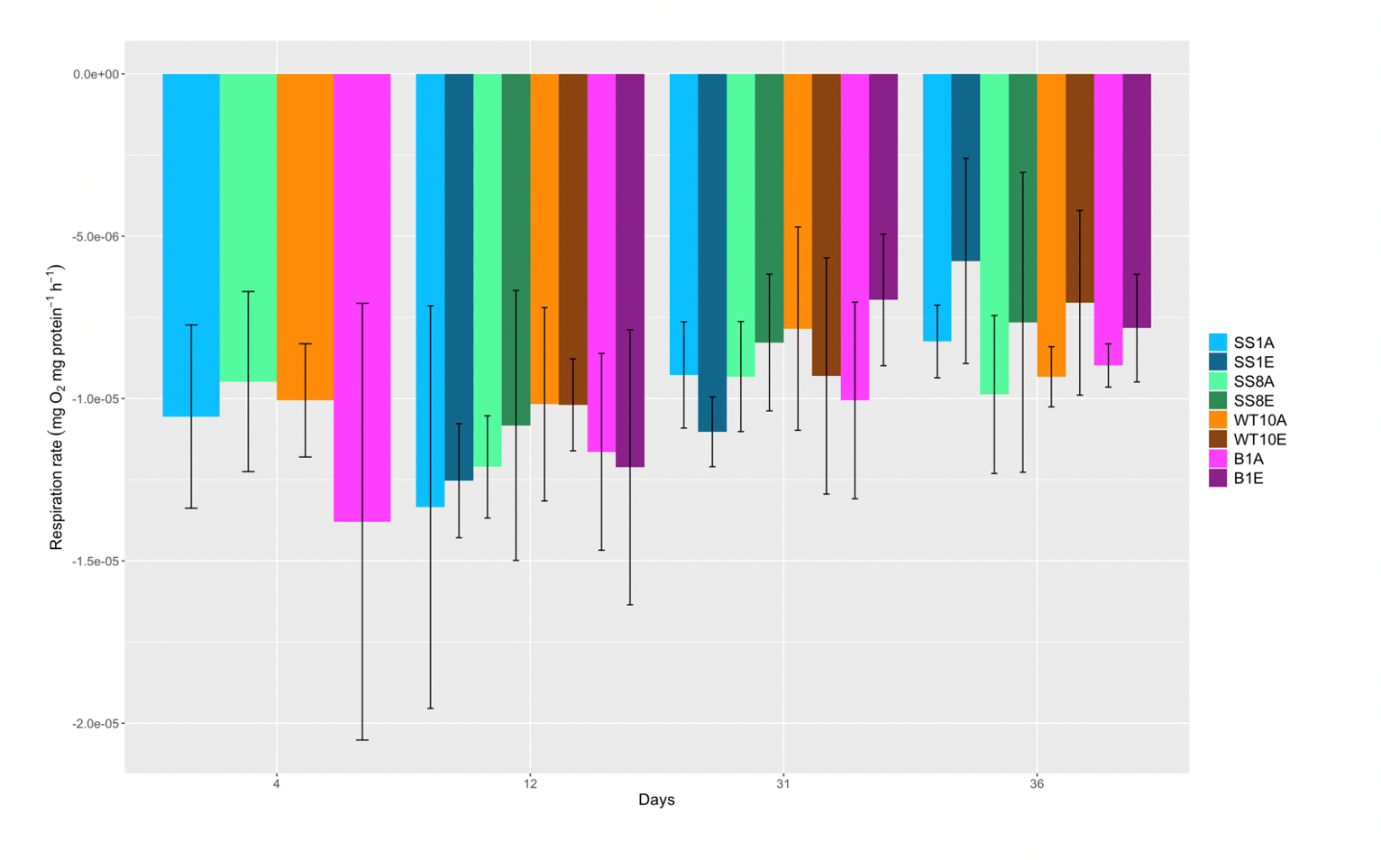


**Figure S5. Respiration rates of anemone groups normalized by host protein content.** A = ambient temperature, E = elevated temperature. Note that no statistically significant difference was detected between temperature treatments within an anemone group. Values are means ± 1SD. Biological replication was n = 4 per anemone groups per temperature per day.


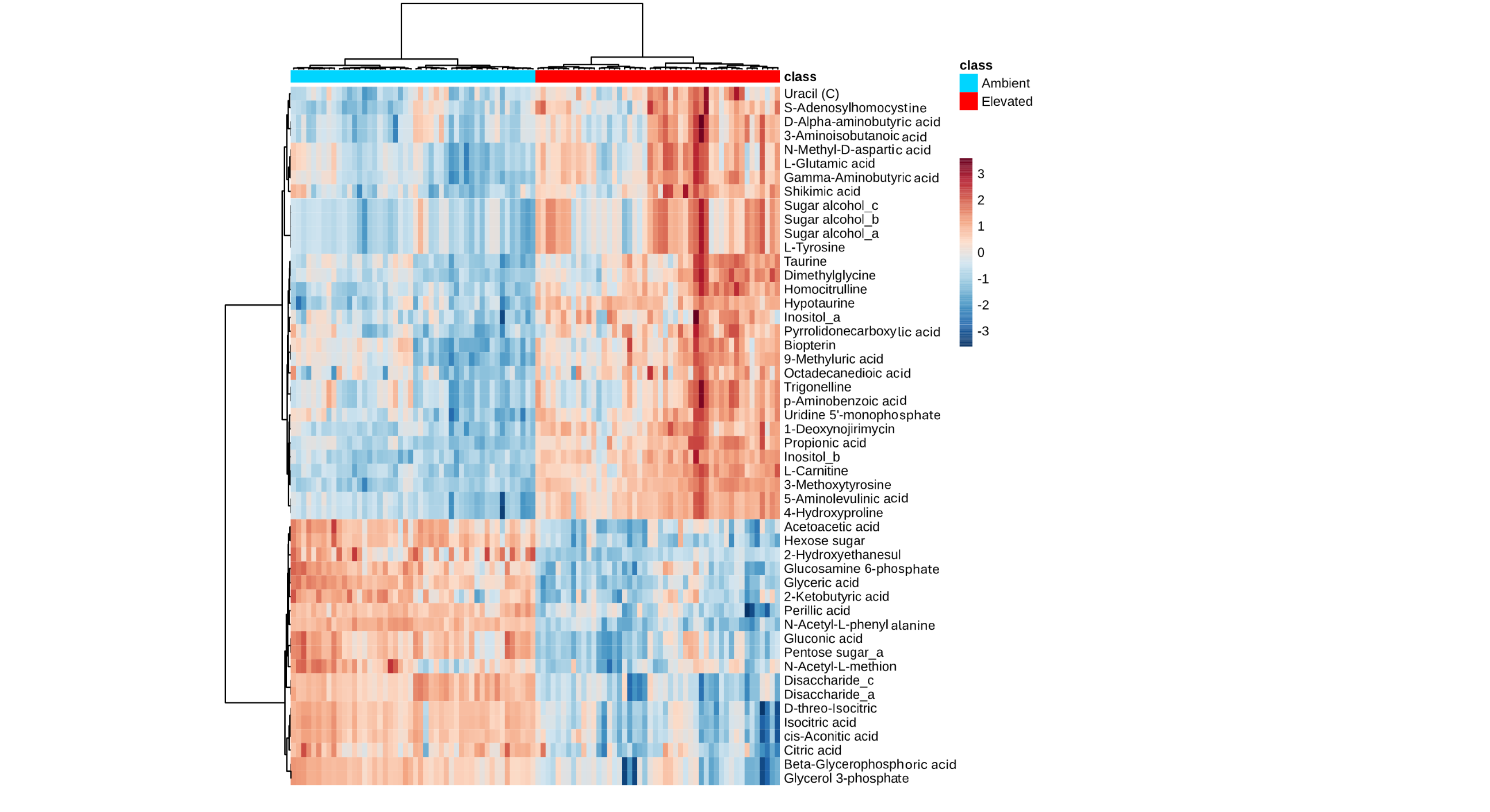


**Figure S6. Heatmap of the top 50 significant metabolites (out of 122)** **of the host fraction of *E. diaphana* between temperature treatments.** Sample and metabolite similarity were based on Euclidean distance and the Ward clustering algorithm. The colour scale refers to log_2_ fold change relative to the mean.

**
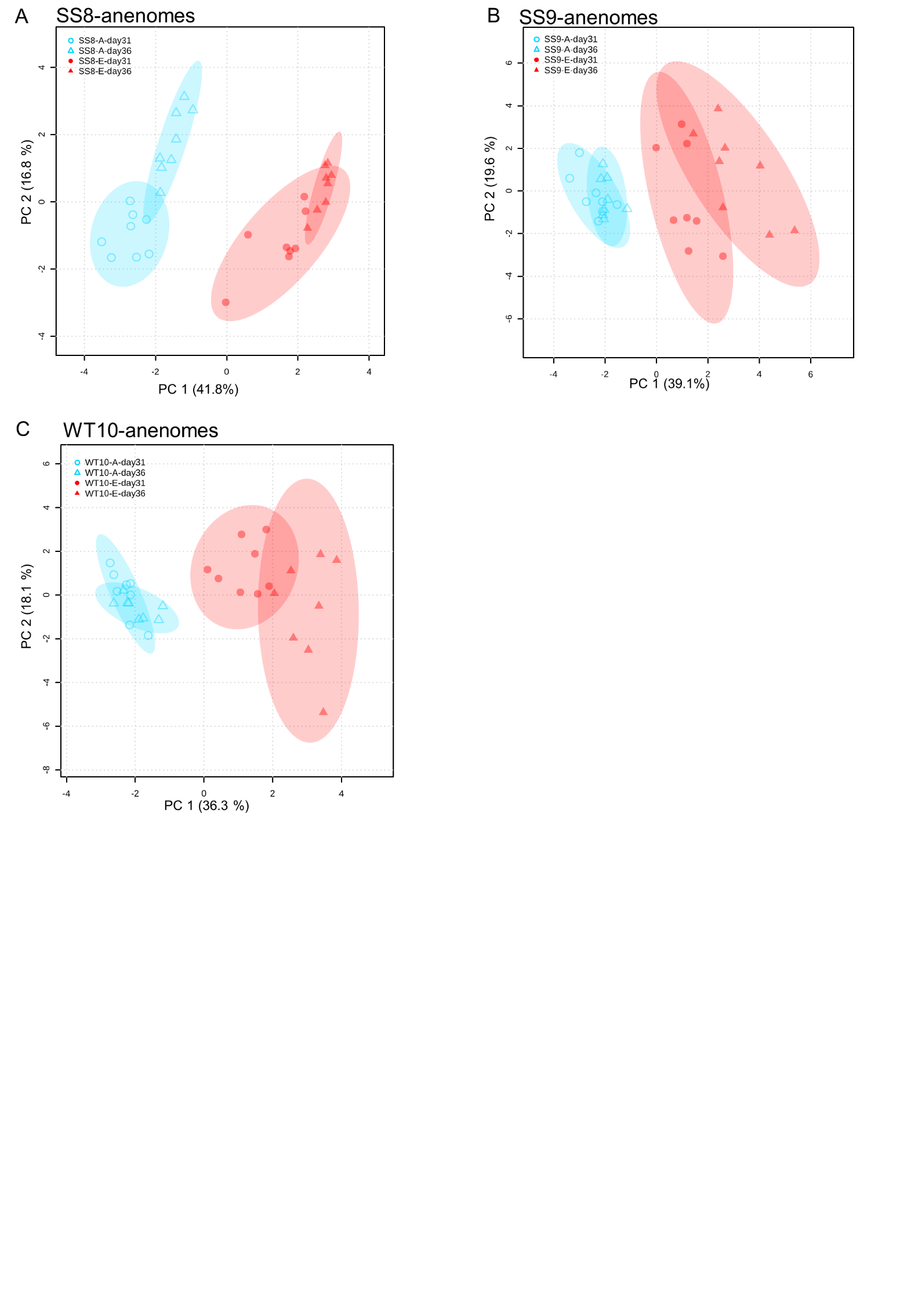
**

**Figure S7. PCA plot of the host fraction of *E. diaphana* using all 184 metabolites for (A) SS8, (B) SS9 and (C) WT10.** Blue and red points represent ambient and elevated temperature treatment, respectively; circle and triangle points represent Day 31 and 36, respectively. A = ambient temperature, E = elevated temperature.

**
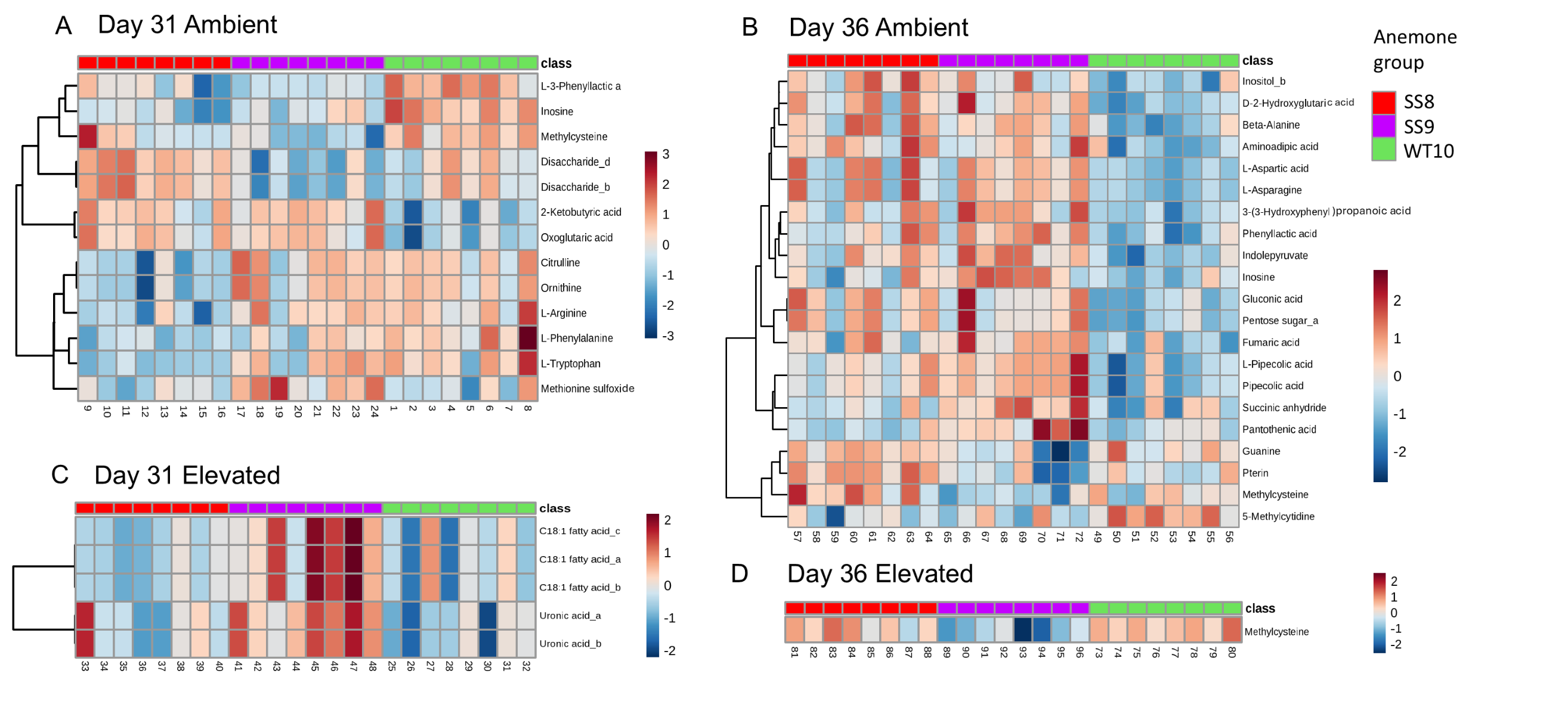
Figure S8. Heatmap of metabolites that were significantly different between *E. diaphana* hosts in the same temperature treatment and on the same sampling day.** (A) Ambient treatment Day 31, (B) ambient treatment Day 36, (C) elevated treatment Day 31, (D) elevated treatment Day 36. Metabolite similarities were calculated based on Euclidean distance and the Ward clustering algorithm. The x-axis is ordered by anemone groups. A = ambient temperature, E = elevated temperature.

**Appendix Tables**

**Table S1. Symbiodiniaceae strains inoculated into the various anemone groups.**

| **Strain** | **Culture ID^a^** | **Strain type** | **Strain character^b^** | **Species** | **°C^c^** | **Original host** | **Source** |
| --- | --- | --- | --- | --- | --- | --- | --- |
| SS1 | SCF 055-01.01 | Heterologous | Heat-evolved, conferring | *Cladocopium proliferum* | 31 °C | *Acropora tenuis* | Magnetic Island |
| SS7 | SCF 055-01.07 | Heterologous | Heat-evolved, conferring | *Cladocopium proliferum* | 31 °C | *Acropora tenuis* | Magnetic Island |
| SS8 | SCF 055-01.08 | Heterologous | Heat-evolved, conferring | *Cladocopium proliferum* | 31 °C | *Acropora tenuis* | Magnetic Island |
| SS3 | SCF 055-01.03 | Heterologous | Heat-evolved, non-conferring | *Cladocopium proliferum* | 31 °C | *Acropora tenuis* | Magnetic Island |
| SS5 | SCF 055-01.05 | Heterologous | Heat-evolved, non-conferring | *Cladocopium proliferum* | 31 °C | *Acropora tenuis* | Magnetic Island |
| SS9 | SCF 055-01.09 | Heterologous | Heat-evolved, non-conferring | *Cladocopium proliferum* | 31 °C | *Acropora tenuis* | Magnetic Island |
| WT10 | SCF 055-01.10 | Heterologous | Wild-type | *Cladocopium proliferum* | 27 °C | *Acropora tenuis* | Magnetic Island |
| B1 | SCF 127-01 | Homologous | Wild-type | *Breviolum minutum* | 27 °C | *Exaiptasia diaphana* | Central GBR |

^a^ Culture ID indicates the identification number of the strain at the Australian Institute of Marine Science.

^b^ Heat-evolved strains were derived from the same mother culture as WT10, they were experimentally evolved under an elevated temperature of 31 °C (Chakravarti et al. 2017). “Conferring” strains were those that conferred enhanced bleaching tolerance to *Acropora tenuis* coral larvae, and “non-conferring” strains are those that did not (Buerger et al. 2020).

^c^ indicates the temperature the culture was maintained at before inoculation

**Table S2. Results of the Mann-Whitney Wilcoxon test on the maximum quantum yield of photosystem II (Fv/Fm).** Comparisons were made between temperature treatments for a specific anemone group at one time point. Only statistically significant results are shown below.

| Strain | Day | W | p-value | n (ambient) | n (elevated) |
| --- | --- | --- | --- | --- | --- |
| SS1 | 18 | 121 | 0.004 | 12 | 12 |
|  | 20 | 108 | 0.039 | 12 | 12 |
|  | 22 | 117 | 0.008 | 12 | 12 |
|  | 24 | 144 | <0.001 | 12 | 12 |
|  | 26 | 125 | 0.001 | 12 | 12 |
|  | 28 | 144 | <0.001 | 12 | 12 |
|  | 30 | 144 | <0.001 | 12 | 12 |
|  | 31 | 132 | <0.001 | 12 | 11 |
| SS7 | 26 | 107 | 0.045 | 12 | 12 |
|  | 28 | 132 | <0.001 | 12 | 12 |
|  | 30 | 144 | <0.001 | 12 | 12 |
|  | 31 | 144 | <0.001 | 12 | 12 |
| SS8 | 30 | 144 | <0.001 | 12 | 12 |
|  | 31 | 120 | <0.001 | 12 | 10 |
| SS3 | 26 | 122 | <0.001 | 12 | 11 |
|  | 28 | 132 | <0.001 | 12 | 11 |
|  | 30 | 48 | 0.001 | 12 | 4 |
| SS5 | 18 | 137 | <0.001 | 12 | 12 |
|  | 20 | 132 | <0.001 | 12 | 12 |
|  | 22 | 139 | <0.001 | 12 | 12 |
|  | 24 | 144 | <0.001 | 12 | 12 |
|  | 26 | 113 | 0.017 | 12 | 12 |
|  | 28 | 119 | 0.006 | 12 | 12 |
|  | 30 | 144 | <0.001 | 12 | 12 |
|  | 31 | 108 | <0.001 | 12 | 9 |
| SS9 | 12 | 144 | <0.001 | 12 | 12 |
|  | 14 | 144 | <0.001 | 12 | 12 |
|  | 16 | 144 | <0.001 | 12 | 12 |
|  | 18 | 144 | <0.001 | 12 | 12 |
|  | 20 | 144 | <0.001 | 12 | 12 |
|  | 22 | 144 | <0.001 | 12 | 12 |
|  | 24 | 144 | <0.001 | 12 | 12 |
|  | 26 | 144 | <0.001 | 12 | 12 |
| WT10 | 28 | 144 | <0.001 | 12 | 12 |
|  | 30 | 132 | <0.001 | 12 | 11 |
|  | 31 | 60 | <0.001 | 12 | 5 |
| B1 | 30 | 132 | <0.001 | 12 | 11 |
|  | 31 | 144 | <0.001 | 12 | 12 |

**Table S3. Results of the Mann-Whitney Wilcoxon test on Symbiodiniaceae cell count (data proportion to Day 4).** Comparisons were made between temperatures for a specific anemone group at one time point. Biological replication was n = 4 per anemone groups per temperature per day. * indicates statistical significance between temperature.

| Strain | Day | W | p-value |
| --- | --- | --- | --- |
| SS1 | 12 | 13 | 0.2 |
|  | 31 | 14 | 0.11 |
|  | 36 | 16 | 0.029* |
| SS7 | 12 | 7 | 0.89 |
|  | 31 | 16 | 0.029* |
|  | 36 | 16 | 0.029* |
| SS8 | 12 | 13 | 0.2 |
|  | 31 | 14 | 0.11 |
|  | 36 | 16 | 0.029* |
| SS3 | 12 | 13 | 0.2 |
|  | 31 | 15 | 0.057 |
|  | 36 | 16 | 0.029* |
| SS5 | 12 | 7 | 0.89 |
|  | 31 | 16 | 0.029* |
|  | 36 | 14 | 0.11 |
| SS9 | 12 | 10 | 0.69 |
|  | 31 | 16 | 0.029* |
|  | 36 | 16 | 0.029* |
| WT10 | 12 | 5 | 0.49 |
|  | 31 | 12 | 0.34 |
|  | 36 | 15 | 0.057 |
| B1 | 12 | 8 | 1 |
|  | 31 | 16 | 0.029* |
|  | 36 | 16 | 0.029* |

**Table S4. Statistical analysis of ∆Symbiodiniaceae cell count using generalized liner model (factor ~Strain, family=quasibinomial).** E = elevated temperature. Comparisons were made between anemone groups under elevated temperature treatment on Day 36. Only statistically significant results are shown below.

| **Strain Comparison** | **Estimate** | **Std. Error** | **t value** | **p-value** |
| --- | --- | --- | --- | --- |
| WT10E – SS7E | -0.84 | 0.35 | -2.39 | 0.025 |
| B1E – WT10E | 1.30 | 0.41 | 3.15 | 0.004 |
| B1E – SS8E | 1.11 | 0.42 | 2.64 | 0.014 |

**Table S5. Statistical analysis of gross photosynthesis rate (normalized by Symbiodiniaceae cell count) and respiration rate (normalized by host protein content) using the Mann-Whitney Wilcoxon test.** Comparisons were made between temperature treatments for a specific anemone group at one time point. Biological replication was n = 4 per anemone groups per temperature per day. * indicates statistical significance between temperature.

|  | Strain | Day | W | p-value |
| --- | --- | --- | --- | --- |
| Gross photosynthesis | SS1 | 12 | 11 | 0.49 |
|  |  | 31 | 16 | 0.029* |
|  |  | 36 | 16 | 0.029* |
|  | SS8 | 12 | 6 | 0.69 |
|  |  | 31 | 16 | 0.029* |
|  |  | 36 | 16 | 0.029* |
|  | WT10 | 12 | 16 | 0.029* |
|  |  | 31 | 16 | 0.029* |
|  |  | 36 | 16 | 0.029* |
|  | B1 | 12 | 10 | 0.69 |
|  |  | 31 | 16 | 0.029* |
|  |  | 36 | 16 | 0.029* |
| Respiration | SS1 | 12 | 10 | 0.69 |
|  |  | 31 | 13 | 0.2 |
|  |  | 36 | 4 | 0.34 |
|  | SS8 | 12 | 7 | 0.89 |
|  |  | 31 | 5 | 0.48 |
|  |  | 36 | 4 | 0.34 |
|  | WT10 | 12 | 8 | 1 |
|  |  | 31 | 10 | 0.69 |
|  |  | 36 | 4 | 0.34 |
|  | B1 | 12 | 8 | 1 |
|  |  | 31 | 2 | 0.11 |
|  |  | 36 | 4 | 0.34 |

**Table S6. List of the 117 significant metabolites found from the host fraction of *E. diaphana* between ambient and elevated treatments overall.** A metabolite was considered significant when P_adj_ < 0.05 and when fold change (FC) > 30%. A FC > 1 suggests that this metabolite was more abundant under ambient temperature (e.g., FC 2= 2 times more abundant under ambient temperature), whereas a FC < 1 indicates that it is more abundant under elevated temperature (e.g., FC 0.5 = 2 times more abundant under elevated temperature). Biological replication was n = 48 (all anemone groups, Day 31 and 36 combined) per temperature.

| **Metabolites** |  | **t.stat** | **FDR** | **FC** | **log2(FC)** |
| --- | --- | --- | --- | --- | --- |
| 1-Deoxynojirimycin |  | -13.70 | < 0.001 | 0.12 | -3.08 |
| 4-Hydroxyproline |  | -14.74 | < 0.001 | 0.13 | -2.94 |
| 5-Aminolevulinic acid |  | -14.36 | < 0.001 | 0.14 | -2.84 |
| 3-Methoxytyrosine |  | -19.80 | < 0.001 | 0.21 | -2.28 |
| Inositol_b |  | -18.40 | < 0.001 | 0.22 | -2.19 |
| Hypotaurine |  | -13.86 | < 0.001 | 0.23 | -2.14 |
| L-Tyrosine |  | -9.24 | < 0.001 | 0.27 | -1.91 |
| Sugar alcohol_a |  | -9.28 | < 0.001 | 0.27 | -1.91 |
| Sugar alcohol_b |  | -9.28 | < 0.001 | 0.27 | -1.91 |
| Sugar alcohol_c |  | -9.28 | < 0.001 | 0.27 | -1.91 |
| L-Carnitine |  | -15.88 | < 0.001 | 0.28 | -1.82 |
| 5-Methylcytidine |  | -5.10 | < 0.001 | 0.31 | -1.71 |
| Shikimic acid |  | -7.99 | < 0.001 | 0.32 | -1.66 |
| 9-Methyluric acid |  | -9.71 | < 0.001 | 0.40 | -1.33 |
| Uridine 5'-monophosphate |  | -9.21 | < 0.001 | 0.41 | -1.30 |
| Inosinic acid |  | -4.02 | < 0.001 | 0.44 | -1.20 |
| S-Adenosylhomocysteine |  | -8.52 | < 0.001 | 0.44 | -1.17 |
| Alanylglycine |  | -5.24 | < 0.001 | 0.45 | -1.15 |
| Oxalic acid |  | -5.14 | < 0.001 | 0.46 | -1.11 |
| Propionic acid |  | -13.50 | < 0.001 | 0.47 | -1.10 |
| D-Ribulose 5-phosphate |  | -4.60 | < 0.001 | 0.49 | -1.04 |
| D-Ribose 5-phosphate |  | -4.60 | < 0.001 | 0.49 | -1.04 |
| Dihydroxyacetone |  | -3.74 | 0.001 | 0.49 | -1.03 |
| L-Cysteine |  | -6.68 | < 0.001 | 0.49 | -1.03 |
| Homocitrulline |  | -10.95 | < 0.001 | 0.50 | -1.01 |
| Biopterin |  | -7.09 | < 0.001 | 0.50 | -1.00 |
| Glycine |  | -5.86 | < 0.001 | 0.50 | -0.99 |
| Octadecanedioic acid |  | -7.53 | < 0.001 | 0.53 | -0.92 |
| Methylcysteine |  | -6.07 | < 0.001 | 0.54 | -0.89 |
| Phosphoric acid (B) |  | -4.81 | < 0.001 | 0.55 | -0.85 |
| L-Threonine |  | -3.65 | 0.001 | 0.55 | -0.85 |
| L-Allothreonine |  | -3.67 | 0.001 | 0.56 | -0.85 |
| L-Cystine |  | -5.39 | < 0.001 | 0.56 | -0.84 |
| L-Homoserine |  | -3.44 | 0.001 | 0.56 | -0.83 |
| N-Methyl-D-aspartic acid |  | -7.64 | < 0.001 | 0.57 | -0.82 |
| L-Glutamic acid |  | -7.66 | < 0.001 | 0.57 | -0.82 |
| Gamma-Aminobutyric acid |  | -10.06 | < 0.001 | 0.57 | -0.81 |
| 3-Aminoisobutanoic acid |  | -6.96 | < 0.001 | 0.57 | -0.80 |
| Caprylic acid |  | -4.67 | < 0.001 | 0.58 | -0.79 |
| D-Alpha-aminobutyric acid |  | -6.79 | < 0.001 | 0.58 | -0.79 |
| Uric acid |  | -5.62 | < 0.001 | 0.58 | -0.79 |
| Glucosamine |  | -6.29 | < 0.001 | 0.58 | -0.78 |
| Inositol_a |  | -7.87 | < 0.001 | 0.58 | -0.78 |
| Pentose sugar_b |  | -5.32 | < 0.001 | 0.59 | -0.75 |
| Pelargonic acid/1-nonaoic acid |  | -3.09 | 0.004 | 0.59 | -0.75 |
| Guanine |  | -6.17 | < 0.001 | 0.59 | -0.75 |
| 2-Methylbutyrylglycine |  | -5.63 | < 0.001 | 0.60 | -0.74 |
| Aminoadipic acid |  | -5.98 | < 0.001 | 0.60 | -0.74 |
| Taurine |  | -8.01 | < 0.001 | 0.60 | -0.74 |
| Cytidine monophosphate |  | -3.44 | 0.001 | 0.60 | -0.74 |
| L-Methionine |  | -5.30 | < 0.001 | 0.60 | -0.74 |
| Ornithine |  | -5.74 | < 0.001 | 0.60 | -0.73 |
| Citrulline |  | -5.45 | < 0.001 | 0.60 | -0.73 |
| Heptadecanoic acid |  | -4.11 | < 0.001 | 0.61 | -0.72 |
| L-Lysine |  | -2.60 | 0.015 | 0.62 | -0.70 |
| Capric acid |  | -3.54 | 0.001 | 0.63 | -0.67 |
| Guanosine monophosphate |  | -4.37 | < 0.001 | 0.63 | -0.67 |
| Cytidine |  | -3.67 | 0.001 | 0.64 | -0.65 |
| L-Asparagine |  | -6.09 | < 0.001 | 0.64 | -0.65 |
| L-Aspartic acid |  | -6.09 | < 0.001 | 0.64 | -0.65 |
| p-Aminobenzoic acid |  | -6.77 | < 0.001 | 0.64 | -0.65 |
| Trigonelline |  | -6.76 | < 0.001 | 0.64 | -0.65 |
| Pyrrolidonecarboxylic acid/pyroglutamic acid |  | -8.30 | < 0.001 | 0.64 | -0.64 |
| Pentadecanoic acid |  | -5.81 | < 0.001 | 0.64 | -0.63 |
| L-Pipecolic acid |  | -5.04 | < 0.001 | 0.65 | -0.63 |
| Pipecolic acid |  | -5.03 | < 0.001 | 0.65 | -0.63 |
| N-Acetyl-D-glucosamine |  | -6.14 | < 0.001 | 0.65 | -0.63 |
| Rhamnose (NPS) |  | -4.80 | < 0.001 | 0.66 | -0.59 |
| Uridine |  | -5.97 | < 0.001 | 0.68 | -0.56 |
| Uracil (C) |  | -7.41 | < 0.001 | 0.68 | -0.55 |
| Stearic acid |  | -2.64 | 0.014 | 0.69 | -0.54 |
| 3-Methylglutaconic acid |  | -2.17 | 0.042 | 0.69 | -0.53 |
| Betaine |  | -6.20 | < 0.001 | 0.71 | -0.50 |
| Succinic acid semialdehyde (MH2) |  | -6.45 | < 0.001 | 0.71 | -0.50 |
| Xanthine |  | -4.64 | < 0.001 | 0.71 | -0.49 |
| 2-Methylglutaric acid |  | -2.72 | 0.011 | 0.72 | -0.47 |
| Adipic acid |  | -2.72 | 0.011 | 0.72 | -0.47 |
| Glycyl-glycine |  | -4.80 | < 0.001 | 0.73 | -0.46 |
| Fructose 6-phosphate |  | -4.68 | < 0.001 | 0.73 | -0.45 |
| Glucose 1-phosphate |  | -4.62 | < 0.001 | 0.73 | -0.45 |
| Beta-Leucine |  | -4.02 | < 0.001 | 0.75 | -0.42 |
| L-Alloisoleucine |  | -4.01 | < 0.001 | 0.75 | -0.42 |
| Maleic acid |  | -2.12 | 0.046 | 0.75 | -0.42 |
| L-Isoleucine |  | -3.96 | < 0.001 | 0.75 | -0.42 |
| N-Acetyl-L-alanine |  | 4.53 | < 0.001 | 1.39 | 0.48 |
| Fumaric acid |  | 4.83 | < 0.001 | 1.42 | 0.50 |
| Deoxyguanosine |  | 4.09 | < 0.001 | 1.46 | 0.54 |
| L-Malic acid |  | 4.83 | < 0.001 | 1.47 | 0.55 |
| Malic acid |  | 4.97 | < 0.001 | 1.49 | 0.58 |
| L-Glutamine |  | 5.42 | < 0.001 | 1.52 | 0.60 |
| Myristic acid |  | 4.91 | < 0.001 | 1.53 | 0.61 |
| Pyruvic acid (MH) |  | 5.18 | < 0.001 | 1.53 | 0.62 |
| N-Acetyl-L-methionine |  | 6.76 | < 0.001 | 1.61 | 0.68 |
| Deoxycytidine |  | 5.68 | < 0.001 | 1.88 | 0.91 |
| Citric acid |  | 7.61 | < 0.001 | 1.91 | 0.93 |
| Glucosamine 6-phosphate |  | 10.30 | < 0.001 | 1.91 | 0.94 |
| Acetoacetic acid |  | 10.33 | < 0.001 | 1.94 | 0.95 |
| dCMP |  | 4.75 | < 0.001 | 1.97 | 0.98 |
| 2-Ketobutyric acid |  | 7.54 | < 0.001 | 2.01 | 1.01 |
| Oxoglutaric acid |  | 6.71 | < 0.001 | 2.06 | 1.05 |
| Thymine |  | 6.34 | < 0.001 | 2.13 | 1.09 |
| 2'-Deoxyguanosine 5'-monophosphate |  | 4.50 | < 0.001 | 2.33 | 1.22 |
| Beta-Glycerophosphoric acid |  | 8.65 | < 0.001 | 2.36 | 1.24 |
| Glycerol 3-phosphate |  | 8.65 | < 0.001 | 2.36 | 1.24 |
| Gluconic acid |  | 10.69 | < 0.001 | 2.59 | 1.37 |
| Pentose sugar_a |  | 11.34 | < 0.001 | 2.62 | 1.39 |
| cis-Aconitic acid |  | 11.06 | < 0.001 | 2.77 | 1.47 |
| Deoxyadenosine monophosphate |  | 5.26 | < 0.001 | 3.00 | 1.59 |
| Isocitric acid |  | 11.98 | < 0.001 | 3.24 | 1.70 |
| Hexose sugar |  | 13.81 | < 0.001 | 3.35 | 1.75 |
| Glyceric acid |  | 16.70 | < 0.001 | 3.40 | 1.77 |
| D-threo-Isocitric acid |  | 11.07 | < 0.001 | 3.45 | 1.79 |
| Perillic acid |  | 11.14 | < 0.001 | 3.83 | 1.94 |
| Disaccharide_c |  | 12.73 | < 0.001 | 6.01 | 2.59 |
| Disaccharide_a |  | 12.83 | < 0.001 | 6.13 | 2.62 |
| 2-Hydroxyethanesulfonate |  | 11.51 | < 0.001 | 6.14 | 2.62 |
| N-Acetyl-L-phenylalanine |  | 20.09 | < 0.001 | 18.61 | 4.22 |

**Table S7. List of the 107 significant metabolites between temperature treatments in the host fraction of SS8-anemones (ANOVA).** Biological replication was n = 16 (Day 31 and 36 combined) per temperature.

| Metabolites | t.stat | FDR | FC | log2(FC) |
| --- | --- | --- | --- | --- |
| 1-Deoxynojirimycin | -8.59 | < 0.001 | 0.11 | -3.14 |
| Hypotaurine | -11.31 | < 0.001 | 0.14 | -2.83 |
| 4-Hydroxyproline | -7.89 | < 0.001 | 0.14 | -2.83 |
| 5-Aminolevulinic acid | -7.70 | < 0.001 | 0.16 | -2.68 |
| 3-Methoxytyrosine | -13.70 | < 0.001 | 0.18 | -2.44 |
| 5-Methylcytidine | -4.30 | < 0.001 | 0.22 | -2.21 |
| Inositol_b | -9.56 | < 0.001 | 0.24 | -2.07 |
| Sugar alcohol_a | -8.46 | < 0.001 | 0.26 | -1.97 |
| Sugar alcohol_b | -8.46 | < 0.001 | 0.26 | -1.97 |
| Sugar alcohol_c | -8.46 | < 0.001 | 0.26 | -1.97 |
| L-Tyrosine | -8.44 | < 0.001 | 0.26 | -1.96 |
| L-Carnitine | -12.60 | < 0.001 | 0.26 | -1.94 |
| Shikimic acid | -4.63 | < 0.001 | 0.28 | -1.85 |
| L-Cysteine | -7.19 | < 0.001 | 0.35 | -1.50 |
| Alanylglycine | -4.61 | < 0.001 | 0.37 | -1.45 |
| 9-Methyluric acid | -5.21 | < 0.001 | 0.40 | -1.32 |
| S-Adenosylhomocysteine | -6.95 | < 0.001 | 0.42 | -1.27 |
| L-Cystine | -5.93 | < 0.001 | 0.42 | -1.26 |
| Cytidine monophosphate | -3.60 | 0.003 | 0.45 | -1.16 |
| Homocitrulline | -6.51 | < 0.001 | 0.45 | -1.15 |
| Propionic acid | -8.39 | < 0.001 | 0.46 | -1.13 |
| Cytidine | -3.90 | 0.001 | 0.46 | -1.12 |
| Uridine 5'-monophosphate | -4.51 | < 0.001 | 0.46 | -1.11 |
| Glycine | -5.23 | < 0.001 | 0.47 | -1.09 |
| Ornithine | -6.87 | < 0.001 | 0.48 | -1.06 |
| D-Ribulose 5-phosphate | -2.50 | 0.031 | 0.49 | -1.03 |
| D-Ribose 5-phosphate | -2.50 | 0.031 | 0.49 | -1.03 |
| Citrulline | -6.23 | < 0.001 | 0.49 | -1.02 |
| Biopterin | -4.10 | 0.001 | 0.50 | -1.01 |
| Phosphoric acid (B) | -4.50 | < 0.001 | 0.50 | -1.00 |
| Dimethylglycine | -4.87 | < 0.001 | 0.51 | -0.97 |
| Gamma-Aminobutyric acid | -6.70 | < 0.001 | 0.53 | -0.90 |
| Methylcysteine | -3.51 | 0.003 | 0.54 | -0.89 |
| L-Threonine | -3.19 | 0.007 | 0.54 | -0.88 |
| L-Allothreonine | -3.23 | 0.007 | 0.54 | -0.88 |
| 3-Aminoisobutanoic acid | -6.84 | < 0.001 | 0.55 | -0.86 |
| L-Homoserine | -3.03 | 0.011 | 0.55 | -0.86 |
| N-Methyl-D-aspartic acid | -4.75 | < 0.001 | 0.55 | -0.86 |
| L-Glutamic acid | -4.74 | < 0.001 | 0.55 | -0.85 |
| D-Alpha-aminobutyric acid | -6.64 | < 0.001 | 0.56 | -0.83 |
| Uric acid | -3.68 | 0.002 | 0.58 | -0.78 |
| L-Lysine | -2.94 | 0.013 | 0.58 | -0.78 |
| Glucosamine | -3.94 | 0.001 | 0.59 | -0.76 |
| Glycyl-glycine | -7.30 | < 0.001 | 0.59 | -0.75 |
| p-Aminobenzoic acid | -4.90 | < 0.001 | 0.60 | -0.75 |
| Trigonelline | -4.89 | < 0.001 | 0.60 | -0.75 |
| Taurine | -4.57 | < 0.001 | 0.60 | -0.74 |
| Oxalic acid | -2.72 | 0.020 | 0.60 | -0.73 |
| Inositol_a | -4.05 | 0.001 | 0.61 | -0.71 |
| Uridine | -5.55 | < 0.001 | 0.61 | -0.70 |
| Pentose sugar_b | -4.30 | < 0.001 | 0.62 | -0.68 |
| L-Methionine | -4.32 | < 0.001 | 0.64 | -0.65 |
| Pipecolic acid | -4.46 | < 0.001 | 0.65 | -0.61 |
| L-Pipecolic acid | -4.45 | < 0.001 | 0.65 | -0.61 |
| Octadecanedioic acid | -2.80 | 0.017 | 0.66 | -0.61 |
| Uracil (C) | -5.26 | < 0.001 | 0.66 | -0.61 |
| Guanine | -3.01 | 0.011 | 0.66 | -0.59 |
| L-Asparagine | -3.77 | 0.002 | 0.67 | -0.59 |
| L-Aspartic acid | -3.77 | 0.002 | 0.67 | -0.59 |
| Pyrrolidonecarboxylic acid/pyroglutamic acid | -4.77 | < 0.001 | 0.67 | -0.59 |
| 2-Methylbutyrylglycine | -2.80 | 0.017 | 0.67 | -0.57 |
| Pantothenic acid | -3.61 | 0.003 | 0.68 | -0.56 |
| Aminoadipic acid | -2.67 | 0.022 | 0.68 | -0.55 |
| Succinic acid semialdehyde (MH2) | -3.53 | 0.003 | 0.73 | -0.46 |
| L-Alloisoleucine | -4.01 | 0.001 | 0.73 | -0.45 |
| Beta-Leucine | -4.00 | 0.001 | 0.73 | -0.45 |
| L-Isoleucine | -3.97 | 0.001 | 0.74 | -0.44 |
| Sarcosine | -2.45 | 0.035 | 0.74 | -0.43 |
| L-Alanine | -2.45 | 0.035 | 0.74 | -0.43 |
| N-Acetyl-D-glucosamine | -2.50 | 0.031 | 0.75 | -0.41 |
| Betaine | -2.88 | 0.014 | 0.76 | -0.40 |
| Rhamnose (NPS) | -2.33 | 0.044 | 0.77 | -0.38 |
| D-2-Hydroxyglutaric acid | 2.66 | 0.023 | 1.31 | 0.39 |
| Fumaric acid | 2.76 | 0.018 | 1.35 | 0.43 |
| N-Acetyl-L-alanine | 2.69 | 0.021 | 1.40 | 0.49 |
| L-Malic acid | 2.76 | 0.018 | 1.41 | 0.50 |
| Malic acid | 2.86 | 0.015 | 1.44 | 0.53 |
| Glycerol | 3.64 | 0.003 | 1.47 | 0.56 |
| Pyruvic acid (MH) | 2.90 | 0.014 | 1.47 | 0.56 |
| N-Acetyl-L-methionine | 3.78 | 0.002 | 1.54 | 0.63 |
| Alpha-Linolenic acid | 3.41 | 0.004 | 1.61 | 0.68 |
| Deoxycytidine | 3.57 | 0.003 | 1.81 | 0.85 |
| Glucosamine 6-phosphate | 5.99 | < 0.001 | 1.91 | 0.94 |
| Acetoacetic acid | 6.06 | < 0.001 | 1.92 | 0.94 |
| Threonic acid | 3.31 | 0.005 | 1.97 | 0.98 |
| Myristic acid | 5.02 | < 0.001 | 2.02 | 1.01 |
| 2-Ketobutyric acid | 4.93 | < 0.001 | 2.04 | 1.03 |
| Thymine | 3.58 | 0.003 | 2.08 | 1.06 |
| dCMP | 3.03 | 0.011 | 2.10 | 1.07 |
| Oxoglutaric acid | 4.70 | < 0.001 | 2.13 | 1.09 |
| Citric acid | 6.76 | < 0.001 | 2.19 | 1.13 |
| Beta-Glycerophosphoric acid | 5.69 | < 0.001 | 2.25 | 1.17 |
| Glycerol 3-phosphate | 5.69 | < 0.001 | 2.25 | 1.17 |
| 2'-Deoxyguanosine 5'-monophosphate | 2.93 | 0.013 | 2.37 | 1.24 |
| Gluconic acid | 5.92 | < 0.001 | 2.37 | 1.25 |
| Pentose sugar_a | 6.40 | < 0.001 | 2.47 | 1.30 |
| cis-Aconitic acid | 8.85 | < 0.001 | 2.71 | 1.44 |
| Deoxyadenosine monophosphate | 2.51 | 0.031 | 3.04 | 1.60 |
| Isocitric acid | 8.72 | < 0.001 | 3.09 | 1.63 |
| D-threo-Isocitric acid | 8.62 | < 0.001 | 3.18 | 1.67 |
| Hexose sugar | 8.61 | < 0.001 | 3.28 | 1.71 |
| Glyceric acid | 10.30 | < 0.001 | 3.68 | 1.88 |
| Perillic acid | 10.68 | < 0.001 | 4.23 | 2.08 |
| Disaccharide_c | 8.48 | < 0.001 | 4.57 | 2.19 |
| Disaccharide_a | 8.69 | < 0.001 | 4.84 | 2.27 |
| 2-Hydroxyethanesulfonate | 12.89 | < 0.001 | 10.25 | 3.36 |
| N-Acetyl-L-phenylalanine | 12.76 | < 0.001 | 16.23 | 4.02 |

**Table S8. List of 114 significant metabolites found from host fraction of SS9-anemones between temperature treatments (ANOVA).** Biological replication was n = 16 (Day 31 and 36 combined) per temperature.

| **Metabolites** | **t.stat** | **FDR** | **FC** | **log2(FC)** |
| --- | --- | --- | --- | --- |
| 1-Deoxynojirimycin | -7.86 | < 0.001 | 0.11 | -3.20 |
| 4-Hydroxyproline | -8.07 | < 0.001 | 0.13 | -2.96 |
| 5-Aminolevulinic acid | -7.66 | < 0.001 | 0.14 | -2.85 |
| 3-Methoxytyrosine | -13.18 | < 0.001 | 0.18 | -2.46 |
| L-Tyrosine | -5.59 | < 0.001 | 0.21 | -2.24 |
| Sugar alcohol_a | -5.64 | < 0.001 | 0.21 | -2.23 |
| Sugar alcohol_b | -5.64 | < 0.001 | 0.21 | -2.23 |
| Sugar alcohol_c | -5.64 | < 0.001 | 0.21 | -2.23 |
| L-Carnitine | -10.65 | < 0.001 | 0.24 | -2.09 |
| Inositol_b | -11.36 | < 0.001 | 0.25 | -1.98 |
| Hypotaurine | -6.83 | < 0.001 | 0.26 | -1.95 |
| Shikimic acid | -4.99 | < 0.001 | 0.31 | -1.68 |
| 9-Methyluric acid | -6.88 | < 0.001 | 0.35 | -1.53 |
| Uridine 5'-monophosphate | -5.23 | < 0.001 | 0.37 | -1.44 |
| Inosinic acid | -3.63 | 0.003 | 0.38 | -1.39 |
| Alanylglycine | -4.02 | 0.001 | 0.39 | -1.36 |
| S-Adenosylhomocysteine | -4.79 | < 0.001 | 0.42 | -1.25 |
| Octadecanedioic acid | -5.25 | < 0.001 | 0.43 | -1.21 |
| Glycine | -3.08 | 0.010 | 0.45 | -1.15 |
| Dimethylglycine | -5.30 | < 0.001 | 0.46 | -1.12 |
| Guanine | -5.32 | < 0.001 | 0.46 | -1.11 |
| D-Ribose 5-phosphate | -3.12 | 0.009 | 0.46 | -1.11 |
| D-Ribulose 5-phosphate | -3.12 | 0.009 | 0.46 | -1.11 |
| Biopterin | -5.40 | < 0.001 | 0.46 | -1.11 |
| Homocitrulline | -6.66 | < 0.001 | 0.47 | -1.08 |
| Pelargonic acid/1-nonaoic acid | -2.59 | 0.026 | 0.49 | -1.03 |
| Uric acid | -4.69 | < 0.001 | 0.50 | -1.01 |
| Caprylic acid | -3.68 | 0.003 | 0.50 | -1.01 |
| Propionic acid | -6.96 | < 0.001 | 0.50 | -1.00 |
| Oxalic acid | -2.61 | 0.025 | 0.51 | -0.98 |
| 2-Methylbutyrylglycine | -4.25 | 0.001 | 0.51 | -0.97 |
| 3-Aminoisobutanoic acid | -4.37 | 0.001 | 0.51 | -0.96 |
| D-Alpha-aminobutyric acid | -4.28 | 0.001 | 0.51 | -0.96 |
| Phosphoric acid (B) | -2.69 | 0.021 | 0.52 | -0.95 |
| Pentadecanoic acid | -4.77 | < 0.001 | 0.54 | -0.90 |
| Heptadecanoic acid | -3.48 | 0.004 | 0.54 | -0.89 |
| Capric acid | -2.86 | 0.016 | 0.54 | -0.88 |
| N-Methyl-D-aspartic acid | -5.13 | < 0.001 | 0.55 | -0.88 |
| L-Glutamic acid | -5.12 | < 0.001 | 0.55 | -0.87 |
| L-Cysteine | -2.96 | 0.013 | 0.55 | -0.87 |
| Maleic acid | -3.55 | 0.004 | 0.56 | -0.84 |
| Gamma-Aminobutyric acid | -6.01 | < 0.001 | 0.56 | -0.83 |
| Taurine | -4.93 | < 0.001 | 0.56 | -0.83 |
| Glucosamine | -4.43 | < 0.001 | 0.57 | -0.82 |
| Methylcysteine | -4.33 | 0.001 | 0.58 | -0.80 |
| 2-Methylglutaric acid | -3.35 | 0.006 | 0.58 | -0.77 |
| Adipic acid | -3.34 | 0.006 | 0.58 | -0.77 |
| Pentose sugar_b | -2.61 | 0.025 | 0.59 | -0.75 |
| Pterin | -3.65 | 0.003 | 0.60 | -0.74 |
| Citrulline | -2.54 | 0.029 | 0.60 | -0.73 |
| N-Acetyl-D-glucosamine | -3.68 | 0.003 | 0.60 | -0.73 |
| L-Methionine | -2.52 | 0.030 | 0.60 | -0.73 |
| L-Cystine | -2.33 | 0.043 | 0.62 | -0.70 |
| Ornithine | -2.44 | 0.035 | 0.62 | -0.69 |
| Xanthine | -3.42 | 0.005 | 0.62 | -0.69 |
| Guanosine monophosphate | -2.39 | 0.038 | 0.62 | -0.68 |
| Uracil (C) | -5.09 | < 0.001 | 0.63 | -0.66 |
| Uridine | -3.35 | 0.006 | 0.63 | -0.66 |
| L-Aspartic acid | -3.27 | 0.007 | 0.63 | -0.66 |
| L-Asparagine | -3.27 | 0.007 | 0.63 | -0.66 |
| Aminoadipic acid | -2.43 | 0.035 | 0.64 | -0.65 |
| Citraconic acid | -3.30 | 0.006 | 0.64 | -0.64 |
| Betaine | -4.19 | 0.001 | 0.65 | -0.63 |
| Mesaconic acid | -3.24 | 0.007 | 0.65 | -0.61 |
| Stearic acid | -2.39 | 0.038 | 0.66 | -0.61 |
| p-Aminobenzoic acid | -3.12 | 0.009 | 0.66 | -0.60 |
| Trigonelline | -3.12 | 0.009 | 0.66 | -0.60 |
| Dimethylmalonic acid | -2.80 | 0.018 | 0.67 | -0.58 |
| Succinic acid semialdehyde (MH2) | -4.03 | 0.001 | 0.67 | -0.58 |
| 3,5-Dihydroxybenzoic acid | -2.81 | 0.017 | 0.68 | -0.57 |
| Pyrrolidonecarboxylic acid/pyroglutamic acid | -4.26 | 0.001 | 0.68 | -0.57 |
| C18:1 fatty acid_a | -2.71 | 0.021 | 0.68 | -0.56 |
| C18:1 fatty acid_c | -2.71 | 0.021 | 0.68 | -0.56 |
| C18:1 fatty acid_b | -2.70 | 0.021 | 0.68 | -0.56 |
| Beta-Leucine | -2.52 | 0.030 | 0.70 | -0.52 |
| L-Alloisoleucine | -2.51 | 0.030 | 0.70 | -0.52 |
| Inositol_a | -3.14 | 0.009 | 0.70 | -0.52 |
| L-Isoleucine | -2.49 | 0.031 | 0.70 | -0.52 |
| Fructose 6-phosphate | -2.64 | 0.024 | 0.71 | -0.48 |
| Glucose 1-phosphate | -2.59 | 0.026 | 0.72 | -0.48 |
| Glyceraldehyde | -2.84 | 0.017 | 0.75 | -0.41 |
| D-Lactic acid | -2.72 | 0.021 | 0.76 | -0.40 |
| Dihydroxyacetone | -2.72 | 0.021 | 0.77 | -0.38 |
| Itaconic acid | 2.87 | 0.016 | 1.34 | 0.42 |
| D-2-Hydroxyglutaric acid | 2.70 | 0.021 | 1.38 | 0.46 |
| L-Glutamine | 2.89 | 0.015 | 1.53 | 0.62 |
| N-Acetyl-L-methionine | 3.02 | 0.011 | 1.59 | 0.67 |
| Fumaric acid | 3.95 | 0.001 | 1.64 | 0.71 |
| Deoxycytidine | 2.71 | 0.021 | 1.65 | 0.72 |
| L-Malic acid | 3.70 | 0.003 | 1.66 | 0.74 |
| Malic acid | 3.77 | 0.002 | 1.70 | 0.76 |
| Pyruvic acid (MH) | 3.92 | 0.001 | 1.74 | 0.80 |
| Glucosamine 6-phosphate | 5.60 | < 0.001 | 1.80 | 0.85 |
| Thymine | 2.99 | 0.012 | 1.85 | 0.89 |
| Oxoglutaric acid | 5.08 | < 0.001 | 1.92 | 0.94 |
| 2-Ketobutyric acid | 6.16 | < 0.001 | 1.96 | 0.97 |
| Acetoacetic acid | 7.17 | < 0.001 | 2.12 | 1.09 |
| Beta-Glycerophosphoric acid | 4.75 | < 0.001 | 2.33 | 1.22 |
| Glycerol 3-phosphate | 4.75 | < 0.001 | 2.33 | 1.22 |
| Citric acid | 5.10 | < 0.001 | 2.52 | 1.33 |
| 2'-Deoxyguanosine 5'-monophosphate | 2.28 | 0.047 | 2.64 | 1.40 |
| 2-Hydroxyethanesulfonate | 5.38 | < 0.001 | 2.66 | 1.41 |
| cis-Aconitic acid | 5.71 | < 0.001 | 2.91 | 1.54 |
| Deoxyadenosine monophosphate | 3.09 | 0.010 | 2.97 | 1.57 |
| Gluconic acid | 7.29 | < 0.001 | 2.97 | 1.57 |
| Pentose sugar_a | 7.84 | < 0.001 | 2.98 | 1.58 |
| Glyceric acid | 10.42 | < 0.001 | 3.25 | 1.70 |
| Perillic acid | 6.49 | < 0.001 | 3.53 | 1.82 |
| Isocitric acid | 7.55 | < 0.001 | 4.40 | 2.14 |
| Hexose sugar | 10.77 | < 0.001 | 4.60 | 2.20 |
| D-threo-Isocitric acid | 7.23 | < 0.001 | 4.83 | 2.27 |
| Disaccharide_c | 6.94 | < 0.001 | 6.26 | 2.65 |
| Disaccharide_a | 6.97 | < 0.001 | 6.35 | 2.67 |
| N-Acetyl-L-phenylalanine | 10.57 | < 0.001 | 14.99 | 3.91 |

**Table S9. List of the 94 significant metabolites found from host fraction of WT10-anemones between temperature treatments (ANOVA).** Biological replication was n = 16 (Day 31 and 36 combined) per temperature.

| **Metabolites** | **t.stat** | **FDR** | **FC** | **log2(FC)** |
| --- | --- | --- | --- | --- |
| 4-Hydroxyproline | -9.98 | < 0.001 | 0.12 | -3.01 |
| 5-Aminolevulinic acid | -9.79 | < 0.001 | 0.12 | -3.00 |
| 1-Deoxynojirimycin | -7.03 | < 0.001 | 0.13 | -2.93 |
| Inositol_b | -11.28 | < 0.001 | 0.17 | -2.54 |
| Disaccharide_d | -2.48 | 0.040 | 0.24 | -2.09 |
| 3-Methoxytyrosine | -8.52 | < 0.001 | 0.26 | -1.94 |
| Hypotaurine | -7.64 | < 0.001 | 0.28 | -1.85 |
| L-Tyrosine | -3.44 | 0.005 | 0.35 | -1.53 |
| Sugar alcohol_a | -3.44 | 0.005 | 0.35 | -1.53 |
| Sugar alcohol_b | -3.44 | 0.005 | 0.35 | -1.53 |
| Sugar alcohol_c | -3.44 | 0.005 | 0.35 | -1.53 |
| Inosinic acid | -3.51 | 0.004 | 0.35 | -1.51 |
| 5-Methylcytidine | -2.72 | 0.024 | 0.35 | -1.50 |
| Oxalic acid | -3.52 | 0.004 | 0.35 | -1.50 |
| L-Carnitine | -6.77 | < 0.001 | 0.38 | -1.40 |
| Shikimic acid | -4.20 | 0.001 | 0.39 | -1.37 |
| Uridine 5'-monophosphate | -6.78 | < 0.001 | 0.40 | -1.31 |
| Propionic acid | -8.15 | < 0.001 | 0.44 | -1.17 |
| 9-Methyluric acid | -4.65 | < 0.001 | 0.46 | -1.13 |
| Inositol_a | -6.84 | < 0.001 | 0.47 | -1.08 |
| Aminoadipic acid | -6.47 | < 0.001 | 0.48 | -1.05 |
| Dimethylglycine | -5.25 | < 0.001 | 0.50 | -0.99 |
| S-Adenosylhomocysteine | -3.76 | 0.003 | 0.50 | -0.99 |
| Methylcysteine | -4.36 | 0.001 | 0.52 | -0.94 |
| Octadecanedioic acid | -5.28 | < 0.001 | 0.52 | -0.93 |
| Rhamnose (NPS) | -4.18 | 0.001 | 0.53 | -0.90 |
| Biopterin | -3.05 | 0.011 | 0.54 | -0.90 |
| Guanosine monophosphate | -3.79 | 0.003 | 0.54 | -0.88 |
| Heptadecanoic acid | -2.42 | 0.044 | 0.56 | -0.85 |
| Pentose sugar_b | -2.78 | 0.021 | 0.57 | -0.82 |
| L-Methionine | -2.86 | 0.018 | 0.57 | -0.81 |
| Homocitrulline | -6.66 | < 0.001 | 0.58 | -0.80 |
| Pyrrolidonecarboxylic acid/pyroglutamic acid | -5.24 | < 0.001 | 0.59 | -0.76 |
| Glucosamine | -2.85 | 0.018 | 0.59 | -0.75 |
| N-Acetyl-D-glucosamine | -4.51 | < 0.001 | 0.60 | -0.73 |
| L-Cysteine | -3.18 | 0.009 | 0.61 | -0.72 |
| N-Methyl-D-aspartic acid | -3.53 | 0.004 | 0.61 | -0.72 |
| L-Glutamic acid | -3.56 | 0.004 | 0.61 | -0.72 |
| Glycine | -2.35 | 0.049 | 0.62 | -0.70 |
| Gamma-Aminobutyric acid | -4.92 | < 0.001 | 0.62 | -0.70 |
| L-Asparagine | -3.63 | 0.004 | 0.62 | -0.69 |
| L-Aspartic acid | -3.63 | 0.004 | 0.62 | -0.69 |
| Pipecolic acid | -3.15 | 0.009 | 0.63 | -0.67 |
| L-Pipecolic acid | -3.14 | 0.009 | 0.63 | -0.67 |
| 2-Methylbutyrylglycine | -2.68 | 0.026 | 0.64 | -0.65 |
| Taurine | -4.31 | 0.001 | 0.64 | -0.63 |
| Pentadecanoic acid | -3.36 | 0.006 | 0.65 | -0.62 |
| p-Aminobenzoic acid | -4.11 | 0.001 | 0.66 | -0.60 |
| Trigonelline | -4.11 | 0.001 | 0.66 | -0.60 |
| Glucose 1-phosphate | -3.11 | 0.010 | 0.67 | -0.58 |
| Guanine | -2.45 | 0.041 | 0.67 | -0.58 |
| Fructose 6-phosphate | -3.08 | 0.010 | 0.67 | -0.57 |
| L-Cystine | -2.57 | 0.033 | 0.67 | -0.57 |
| 3-Aminoisobutanoic acid | -2.39 | 0.047 | 0.68 | -0.56 |
| D-Alpha-aminobutyric acid | -2.35 | 0.049 | 0.68 | -0.55 |
| Glucose 6-phosphate | -3.08 | 0.010 | 0.71 | -0.49 |
| Betaine | -3.65 | 0.004 | 0.72 | -0.46 |
| Succinic acid semialdehyde (MH2) | -3.67 | 0.004 | 0.73 | -0.45 |
| Xanthine | -2.47 | 0.040 | 0.75 | -0.41 |
| Taurocholic acid | 2.51 | 0.037 | 1.32 | 0.40 |
| Citric acid | 2.41 | 0.045 | 1.33 | 0.41 |
| Pyruvic acid (MH) | 2.36 | 0.049 | 1.37 | 0.45 |
| Alpha-Linolenic acid | 3.35 | 0.006 | 1.43 | 0.51 |
| Uronic acid_a | 3.60 | 0.004 | 1.46 | 0.55 |
| Uronic acid_b | 3.66 | 0.004 | 1.47 | 0.56 |
| N-Acetyl-L-alanine | 3.55 | 0.004 | 1.47 | 0.56 |
| Adenine | 3.22 | 0.008 | 1.64 | 0.72 |
| Adenosine | 3.18 | 0.009 | 1.65 | 0.73 |
| N-Acetyl-L-methionine | 5.39 | < 0.001 | 1.71 | 0.77 |
| Acetoacetic acid | 4.91 | < 0.001 | 1.76 | 0.82 |
| L-Glutamine | 6.57 | < 0.001 | 1.89 | 0.92 |
| dCMP | 3.01 | 0.012 | 1.98 | 0.99 |
| 2'-Deoxyguanosine 5'-monophosphate | 2.53 | 0.036 | 2.01 | 1.00 |
| 2-Ketobutyric acid | 3.53 | 0.004 | 2.03 | 1.02 |
| Glucosamine 6-phosphate | 6.53 | < 0.001 | 2.05 | 1.04 |
| Oxoglutaric acid | 3.24 | 0.008 | 2.16 | 1.11 |
| Deoxyguanosine | 3.62 | 0.004 | 2.29 | 1.19 |
| Pentose sugar_a | 6.65 | < 0.001 | 2.38 | 1.25 |
| Deoxycytidine | 4.17 | 0.001 | 2.40 | 1.26 |
| Gluconic acid | 6.58 | < 0.001 | 2.42 | 1.27 |
| Glycerol 3-phosphate | 4.96 | < 0.001 | 2.52 | 1.34 |
| Beta-Glycerophosphoric acid | 4.96 | < 0.001 | 2.52 | 1.34 |
| Hexose sugar | 5.78 | < 0.001 | 2.56 | 1.35 |
| Isocitric acid | 5.75 | < 0.001 | 2.61 | 1.38 |
| Thymine | 4.74 | < 0.001 | 2.69 | 1.43 |
| cis-Aconitic acid | 6.07 | < 0.001 | 2.69 | 1.43 |
| D-threo-Isocitric acid | 5.21 | < 0.001 | 2.83 | 1.50 |
| Deoxyadenosine monophosphate | 3.35 | 0.006 | 3.00 | 1.59 |
| Glyceric acid | 8.98 | < 0.001 | 3.27 | 1.71 |
| Perillic acid | 5.47 | < 0.001 | 3.77 | 1.91 |
| 2-Hydroxyethanesulfonate | 5.96 | < 0.001 | 6.25 | 2.64 |
| Disaccharide_a | 7.36 | < 0.001 | 7.55 | 2.92 |
| Disaccharide_c | 7.38 | < 0.001 | 7.61 | 2.93 |
| N-Acetyl-L-phenylalanine | 11.97 | < 0.001 | 25.94 | 4.70 |

**Table S10. List of the 13 metabolites that were significantly different between anemone groups (host fraction) within ambient treatment on Day 31 (ANOVA).**

|  | **f.value** | **p.value** | **-log10(p)** | **FDR** | **Fisher's LSD** |
| --- | --- | --- | --- | --- | --- |
| Inosine | 17.55 | < 0.001 | 4.48 | 0.006 | WT10-A-day31 - SS8-A-day31; WT10-A-day31 - SS9-A-day31 |
| Disaccharide_b | 14.41 | < 0.001 | 3.94 | 0.008 | SS8-A-day31 - SS9-A-day31; SS8-A-day31 - WT10-A-day31; WT10-A-day31 - SS9-A-day31 |
| Disaccharide_d | 14.00 | < 0.001 | 3.86 | 0.008 | SS8-A-day31 - SS9-A-day31; SS8-A-day31 - WT10-A-day31; WT10-A-day31 - SS9-A-day31 |
| Oxoglutaric acid | 12.52 | < 0.001 | 3.58 | 0.012 | SS8-A-day31 - WT10-A-day31; SS9-A-day31 - WT10-A-day31 |
| Ornithine | 12.03 | < 0.001 | 3.48 | 0.012 | SS9-A-day31 - SS8-A-day31; WT10-A-day31 - SS8-A-day31 |
| 2-Ketobutyric acid | 11.73 | < 0.001 | 3.42 | 0.012 | SS8-A-day31 - WT10-A-day31; SS9-A-day31 - WT10-A-day31 |
| L-3-Phenyllactic acid | 10.85 | 0.001 | 3.24 | 0.015 | WT10-A-day31 - SS8-A-day31; WT10-A-day31 - SS9-A-day31 |
| Citrulline | 10.13 | 0.001 | 3.08 | 0.019 | SS9-A-day31 - SS8-A-day31; WT10-A-day31 - SS8-A-day31 |
| Methylcysteine | 8.81 | 0.002 | 2.78 | 0.034 | SS8-A-day31 - SS9-A-day31; WT10-A-day31 - SS9-A-day31 |
| L-Tryptophan | 8.01 | 0.003 | 2.58 | 0.040 | SS9-A-day31 - SS8-A-day31; WT10-A-day31 - SS8-A-day31 |
| Methionine sulfoxide | 7.99 | 0.003 | 2.58 | 0.040 | SS9-A-day31 - SS8-A-day31; SS9-A-day31 - WT10-A-day31 |
| L-Phenylalanine | 7.86 | 0.003 | 2.55 | 0.040 | WT10-A-day31 - SS8-A-day31; WT10-A-day31 - SS9-A-day31 |
| L-Arginine | 7.84 | 0.003 | 2.54 | 0.040 | SS9-A-day31 - SS8-A-day31; WT10-A-day31 - SS8-A-day31 |

**Table S11. List of the 21 metabolites that were significantly different between anemone groups (host fraction) within ambient treatment on Day 36 (ANOVA).1**

|  | **f.value** | **p.value** | **-log10(p)** | **FDR** | **Fisher's LSD** |
| --- | --- | --- | --- | --- | --- |
| Pipecolic acid | 13.08 | < 0.001 | 3.69 | 0.020 | SS9-A-day36 - SS8-A-day36; SS8-A-day36 - WT10-A-day36; SS9-A-day36 - WT10-A-day36 |
| L-Pipecolic acid | 13.03 | < 0.001 | 3.68 | 0.020 | SS9-A-day36 - SS8-A-day36; SS8-A-day36 - WT10-A-day36; SS9-A-day36 - WT10-A-day36 |
| Pterin | 10.43 | 0.001 | 3.15 | 0.030 | SS8-A-day36 - SS9-A-day36; SS8-A-day36 - WT10-A-day36 |
| Indolepyruvate | 10.27 | 0.001 | 3.11 | 0.030 | SS8-A-day36 - WT10-A-day36; SS9-A-day36 - WT10-A-day36 |
| D-2-Hydroxyglutaric acid | 10.22 | 0.001 | 3.10 | 0.030 | SS8-A-day36 - WT10-A-day36; SS9-A-day36 - WT10-A-day36 |
| Pantothenic acid | 9.80 | 0.001 | 3.01 | 0.031 | SS9-A-day36 - SS8-A-day36; SS9-A-day36 - WT10-A-day36 |
| Phenyllactic acid | 9.01 | 0.001 | 2.83 | 0.040 | SS8-A-day36 - WT10-A-day36; SS9-A-day36 - WT10-A-day36 |
| Methylcysteine | 8.65 | 0.002 | 2.74 | 0.043 | SS8-A-day36 - SS9-A-day36; WT10-A-day36 - SS9-A-day36 |
| Beta-Alanine | 7.92 | 0.003 | 2.56 | 0.044 | SS8-A-day36 - WT10-A-day36; SS9-A-day36 - WT10-A-day36 |
| Aminoadipic acid | 7.76 | 0.003 | 2.52 | 0.044 | SS8-A-day36 - WT10-A-day36; SS9-A-day36 - WT10-A-day36 |
| Inositol_b | 7.56 | 0.003 | 2.47 | 0.044 | SS8-A-day36 - WT10-A-day36; SS9-A-day36 - WT10-A-day36 |
| Pentose sugar_a | 7.38 | 0.004 | 2.43 | 0.044 | SS8-A-day36 - WT10-A-day36; SS9-A-day36 - WT10-A-day36 |
| Succinic anhydride | 7.32 | 0.004 | 2.41 | 0.044 | SS9-A-day36 - SS8-A-day36; SS9-A-day36 - WT10-A-day36 |
| Inosine | 7.26 | 0.004 | 2.40 | 0.044 | SS9-A-day36 - SS8-A-day36; SS9-A-day36 - WT10-A-day36 |
| Fumaric acid | 7.16 | 0.004 | 2.37 | 0.044 | SS9-A-day36 - WT10-A-day36 |
| L-Asparagine | 7.13 | 0.004 | 2.36 | 0.044 | SS8-A-day36 - WT10-A-day36; SS9-A-day36 - WT10-A-day36 |
| L-Aspartic acid | 7.13 | 0.004 | 2.36 | 0.044 | SS8-A-day36 - WT10-A-day36; SS9-A-day36 - WT10-A-day36 |
| Guanine | 7.08 | 0.004 | 2.35 | 0.044 | SS8-A-day36 - SS9-A-day36; WT10-A-day36 - SS9-A-day36 |
| 5-Methylcytidine | 7.03 | 0.005 | 2.34 | 0.044 | WT10-A-day36 - SS8-A-day36; WT10-A-day36 - SS9-A-day36 |
| 3-(3-Hydroxyphenyl)propanoic acid | 6.95 | 0.005 | 2.32 | 0.044 | SS8-A-day36 - WT10-A-day36; SS9-A-day36 - WT10-A-day36 |
| Gluconic acid | 6.83 | 0.005 | 2.29 | 0.045 | SS8-A-day36 - WT10-A-day36; SS9-A-day36 - WT10-A-day36 |

**Table S12. List of the significant metabolites between anemone groups under elevated treatment on Day 31 and 36 (ANOVA, host fraction).**

| **Day** | **Metabolite** | **f.value** | **p.value** | **-log10(p)** | **FDR** | **Fisher's LSD** |
| --- | --- | --- | --- | --- | --- | --- |
| 31 | C18:1 fatty acid_a | 9.55 | 0.001 | 2.95 | 0.046 | SS9-E-day31 - SS8-E-day31; SS9-E-day31 - WT10-E-day31 |
| 31 | C18:1 fatty acid_c | 9.55 | 0.001 | 2.95 | 0.046 | SS9-E-day31 - SS8-E-day31; SS9-E-day31 - WT10-E-day31 |
| 31 | Uronic acid_b | 9.54 | 0.001 | 2.95 | 0.046 | SS9-E-day31 - SS8-E-day31; SS9-E-day31 - WT10-E-day31 |
| 31 | C18:1 fatty acid_b | 9.40 | 0.001 | 2.92 | 0.046 | SS9-E-day31 - SS8-E-day31; SS9-E-day31 - WT10-E-day31 |
| 31 | Uronic acid_a | 9.35 | 0.001 | 2.90 | 0.046 | SS9-E-day31 - SS8-E-day31; SS9-E-day31 - WT10-E-day31 |
| 36 | Methylcysteine | 18.3 | < 0.001 | 4.60 | 0.005 | SS8-E-day36 - SS9-E-day36; WT10-E-day36 - SS9-E-day36 |

**Table S13. List of raw data and R codes of this study (https://doi.org/10.5281/zenodo.13824742).**

| **Category** | **File name** | **Description** |
| --- | --- | --- |
| Symbiodiniaceae community | 2021_Thermal-tolerance-of-sea-anemone_ITS2.docx | R codes |
|  | 2021_Thermal tolerance of sea anemone_ ITS2 analysis for paper.csv | Raw data |
| Photochemical efficiency | 2021_Thermal-tolerance-of-sea-anemone_Photochemical-efficiency.docx | R codes |
|  | 2021_Thermal tolerance of sea anemone_Photochemical efficiency F0 more than 0.1 for paper.csv | Raw data |
| Symbiodiniaceae density | 2021_Thermal-tolerance-of-sea-anemone_Symbiodiniaceae-cell-count.docx | R codes |
|  | 2021_Thermal tolerance of sea anemone_Sym cell count.csv | Raw data |
|  | 2021_Thermal tolerance of sea anemone_Delta Cell count.csv | Raw data |
| Respirometry | 2021_Thermal-tolerance-of-sea-anemone_Respirometry.docx | R codes |
|  | 2021_Thermal tolerance of sea anemone_ Respirometry blank corrected for paper.csv | Raw data |
| Metabolomics | 2021_Thermal tolerance of sea anemone_Host metabolome normalized by dry weight.csv | Raw data |

**Appendix Methods**

**S1. DNA extraction, PCR amplification and library preparation**

Each individual anemone was placed in a 1.5 mL tube filled with 1,000 μL filtered RSW and 100 mg of sterile glass beads (425-600 μm), and homogenized with a bead beater (TissueLyser II, Qiagen, Germany); 30 Hz for 3.5 mins. Then, 250 μL of homogenates were transferred to the other set of tubes and kept under -80 °C until DNA extraction. DNA was extracted using the Wayne’s method, with an extra15 min incubation in 10 mg mL^-1^ lysozyme, and 20 s bead beating at 30 Hz with 100 mg of 425- 600 sterile acid-washed glass beads (Wilson et al. 2002). One extraction blank and one PCR negative control were included. The Symbiodiniaceae-specific ITS2 primers used were; Sym_Var_5.8S2 [5’ GTGACCTATGAACTCAGGAGTCGAATTGCAGAACTCCGTGAACC 3’] (Hume et al. 2015) and Sym_Var_Rev [5’ CTGAGACTTGCACATCGCAGCCGGGTTCWCTTGTYTGACTTCATGC 3’] (Hume et al. 2013), with the Illumina Nextera adapters underlined. The thermocycler parameters were: one cycle of initial denaturation at 95 °C (3 min) and 18 cycles of denaturation at 95 °C (15 s), annealing at 55 °C (30 s), extension at 72 °C (30 s), one cycle of final elongation step at 72 °C (7 min) and a final hold temperature at 4 °C.

For the library preparation, triplicate PCR products were pooled into 20 μL and purified using Ampure XP magnetic beads, followed by the resuspension of the purified DNA and 40 μL of nuclease-free water. Ten μL each of DNA, 10 μL of 2x Tap master mix (M0270S, New England, BioLabs, Australia) and 1 μL each of forward indexing and reverse indexing primers (both at 5 μM) were combined. The thermocycler parameters were; one cycle of initial denaturation at 95 °C (3 min) and 24 cycles of denaturation at 95 °C (15 s), annealing at 60 °C (30 s), extension at 72 °C (30 s), one cycle of extension at 72 °C (7 min) and a final hold temperature at 4 °C. Product size and quality were visualized on 1% agarose 0.5 x TAE agarose gel. Five μL of each reaction was pooled (total volume of 50 μL) followed by a final bead clean-up. Library sequencing was conduction via Illumina MiSeq v3 at Walter and Eliza Hall Institute. Raw sequence data were sent to SymPortal for Symbiodiniaceae community analysis.

**S2. Host protein and Symbiodiniaceae cell density**

Each sample was homogenized in a glass homogeniser filled with 1 mL of filtered RSW (fRSW). A total of 500 μL of the homogenate were centrifuged at 5,000 x g for 5 min to separate the Symbiodiniaceae cells and anemone host tissues. The Symbiodiniaceae fraction (i.e., pellet) was vortexed with 500 μL of fRWS and centrifuged again at 5,000 x g for 5 min. The pallet was resuspended in 500 μL of fRWS and stored at -20 °C; later measured in the aforementioned method. The host fraction (i.e., supernatant) was transferred to another set of tubes and stored at -20 °C until measurement using the Bradford assay (Bradford 1976). In addition to the seven bovine serum albumin (BSA) standards from the Bio-Rad kit (Bio-Rad 500 0207) (0.125, 0.25, 0.5, 0.75, 1, 1.5 and 2 mg mL^-1^), four more standards (0.0125, 0.025, 0.05 and 0.075 mg mL^-1^) were made by diluting the original standard with Nuclease-free water (Dungan et al. 2020). The 11 standards, negative controls (fRSW, Nuclease-free water) and thawed host samples were transferred in triplicates (20 μL each), and 200 μL of Bradford reagent was added to each well. After 30 mins, host protein contents were measured via a plate reader (EnSpire microplate reader MLD2300) at 595 nm absorbance.
